# Addressing knowledge, attitude and practice gaps for effective dengue management strategies in Indonesia

**DOI:** 10.3389/fpubh.2025.1540121

**Published:** 2025-05-23

**Authors:** Ari Prayitno, Mei Neni Sitaresmi, Bachti Alisjahbana, Carolina Halim, Fauchil Wardati, Mentari Yudiansyach, Sri Rezeki Hadinegoro

**Affiliations:** ^1^Faculty of Medicine, Universitas Indonesia, Jakarta, Indonesia; ^2^Faculty of Medicine, Universitas Gadjah Mada, Yogyakarta, Indonesia; ^3^Department of Internal Medicine, Hasan Sadikin General Hospital, Bandung, Indonesia; ^4^Research Center for Care and Control of Infectious Diseases, Universitas Padjadjaran, Bandung, Indonesia; ^5^Medical Affairs, PT Takeda Innovative Medicines, Jakarta, Indonesia

**Keywords:** dengue, vaccine, knowledge, attitude, practice, Indonesia, DHF

## Abstract

**Background:**

Dengue is a significant public health issue in Indonesia, facing a substantial year-round disease burden and rising incidence. However, comprehensive assessments of Knowledge, Attitudes, and Practices (KAP) regarding dengue in the Indonesian adult population are limited. This study provides one of the first comprehensive evaluations of KAP in Indonesia, aiming to inform integrated dengue management programs.

**Methods:**

This study utilized data from the larger GEMKAP study, which covered seven countries, including Indonesia. The GEMKAP study was a cross-sectional electronic survey conducted in September and October 2022, targeting adults aged 21 to 60, recruited through email invitations from an existing web-based panel. The survey, consisting of 35 questions, was developed based on existing dengue KAP studies and was translated into Bahasa Indonesia.

**Results:**

Analysis of 600 Indonesian responses revealed higher levels of Attitudes (65%) and Practices (56%) compared to Knowledge (46%). Most respondents correctly identified dengue transmission through *Aedes* mosquitoes (85%) and mosquito breeding grounds (98%); however, awareness of dengue serotypes (48%) and multiple infection risks (50%) was lower. Out of the dengue prevention methods practiced, draining and covering water containers was rated the safest and most effective (8.4 and 8.1, respectively, on a scale from 0 to 10). In comparison, dengue vaccination was perceived as generally safe and effective (7.6 and 7.7, respectively, on a scale from 0 to 10). Willingness to receive dengue vaccines was moderate (51%), with 60% unaware of vaccine availability. Fear of side effects (18%) was the most common reason for moderate willingness to vaccinate. Respondents preferred search engines (88%) and social media (85%) as sources to search health information, with doctors being the most trusted stakeholder to receive health information from (94%). The most favored dengue management strategy was combining vaccination with education and vector control (42%).

**Conclusion:**

The KAP assessment identified strengths and gaps in dengue awareness and practices among Indonesians. The gaps identified from the KAP results underscore the need for an integrated approach combining vector control, vaccination, and education. As the most trusted stakeholders, HCPs can play a key role in supporting the effective implementation of dengue management strategies.

## 1 Introduction

Dengue is a vector-borne viral disease transmitted through the bite of an infected female *Aedes* mosquito. As one of the most prevalent vector-borne illnesses with increasing incidence of infection from 500,000 to 5.2 million over the past two decades, it poses a significant global public health challenge (1). The disease can be caused by any of the four dengue viruses (DENV) serotypes (DENV-1, 2, 3, 4), which initially manifest as flu-like symptoms, and can progress to more severe and life-threatening forms, including dengue hemorrhagic fever and dengue shock syndrome (2).

Southeast Asia accounts for more than half of the global burden of dengue (1), among which Indonesia stands as a highly endemic country with dengue cases reported year-round. Dengue has spread to almost all provinces, with a particular impact on West Java, Bali, East Kalimantan, and Yogyakarta, affecting both large urban centres and rural communities (3–8). In 2022, the Indonesian Ministry of Health recorded 143,000 reported cases of dengue, accounting for an incidence rate of 52 per 100,000 people (9). However, this figure likely underestimates the true caseload, which may be approximately 40 times higher than the nationally reported rates (10). The underestimation is partly attributed to the underreporting and the prevalence of asymptomatic infections. Only 30% of symptomatic individuals seek medical care, further compounded by misdiagnosis (2, 9). In 2022 alone, Indonesia recorded 1,236 deaths due to dengue, with a significant proportion occurring in children under 14 years of age (9). Additionally, among those infected, approximately 5% develop symptoms of severe dengue (11). Beyond the clinical burden, dengue also imposes a substantial economic burden. A study estimated that the total national cost of dengue illness in Indonesia amounted to US$682 million in 2017, including both direct costs (e.g., medical care costs) and indirect costs (e.g., the economic value of lost productivity). Notably, nearly half of this financial burden was attributed to hospitalized cases (12).

To mitigate the high burden of dengue, the Indonesian Ministry of Health has implemented various prevention and control initiatives, including peri-focal spraying of adult mosquitoes, mass larviciding, and community health education (13). Studies have found that to establish a sustainable and comprehensive dengue management program, an integrated approach including vector control, vaccination, and education tailored to the local context should be considered (14, 15). The Indonesian Ministry of Health has adopted such an integrated strategy through the implementation of the National Dengue Control Strategy 2021–2025 (‘*Strategi Nasional (STRANAS) Penanggulangan Dengue 2021–2025’*). As a part of the integrated dengue prevention and control approach, Indonesia implemented a *Wolbachia* program, which uses naturally occurring *Wolbachia* bacteria to reduce dengue transmission. The program was first piloted in Yogyakarta in 2016 and showed promising results in reducing dengue infection rates (15). Currently, its implementation has been expanded to several provinces in Indonesia (16).

Additionally, dengue vaccines are available in Indonesia as an additional prevention method. Previous modeling studies have shown that the implementation of dengue vaccination is cost-effective, contributing to the reduction of dengue incidence and mortality rates in endemic regions (17). Dengue vaccines have also received recommendations from Indonesian medical associations (18, 19). Currently, dengue vaccines are only accessible through out-of-pocket payment and are not included in the National Immunization Program (NIP). In 2023, a public dengue vaccination program was introduced for primary school children in Balikpapan City, East Kalimantan Province (20). Although education, vector control, and vaccination programs have been implemented in Indonesia, there is still a need to improve the widespread availability of these programs across the archipelago. It is essential to leverage a diverse array of dengue prevention and management tools to effectively protect the population from dengue.

Despite the rising dengue incidence and burden in Indonesia, there have been no national or multi-provincial studies assessing the Knowledge, Attitudes and Practices (KAP) of the general adult population regarding dengue disease and prevention, with only a few province-specific studies available (13, 21). This study is a secondary analysis of data from a larger the Asia Pacific and Latin American Knowledge Attitude Practice (GEMKAP) study which was conducted in 2022 across seven countries in Latin America (Argentina, Brazil, Colombia, Mexico) and Asia Pacific (Indonesia, Malaysia, Singapore) to assess the general population’s KAP levels on dengue, vector control, prevention, and vaccination, including willingness to vaccinate (14). Overall, across the seven countries, global scores (standardized, 0–100% scale) for Knowledge (48%) and Practice (44%) were low, while the Attitude score was moderate (66%) (14). Given Indonesia’s high and persistent dengue burden, a focused analysis of its KAP is crucial to identify gaps and tailor interventions that support national policies. By analyzing Indonesia-specific data from the GEMKAP study, this study aims to derive key messages for stakeholders involved in designing and implementing effective and country-specific integrated dengue management programs.

## 2 Materials and methods

This study was part of the larger study (GEMKAP), which assessed KAP levels on dengue, vector control, prevention, and vaccination, including willingness to vaccinate, across seven countries. To derive Indonesia-specific insights, Indonesian data was extracted from the GEMKAP study for this analysis, with no additional data collection undertaken. To provide local context and validate the GEMKAP findings within the Indonesian setting, four Indonesian experts were consulted. These experts included Internal Medicine physicians and pediatricians specializing in infectious diseases and social pediatrics, and child behavior development. Consultation sessions with the Indonesian experts were conducted virtually, primarily through online meetings. Their input was meticulously incorporated to ensure the study findings are robust, contextually relevant, and accurately reflect the current dengue disease landscape and management practices in Indonesia.

### 2.1 Study design

The GEMKAP study was a cross-sectional, quantitative electronic survey which was conducted between September and October 2022 to assess the KAP regarding dengue disease and vaccines among an adult population in Indonesia. A sample size of 600 for Indonesia was identified as sufficient to infer national representativeness based on a 95% confidence interval and a 5% margin of error. The sampling quota was based on age, household income and region to ensure the sociodemographic representativeness of the national general adult population. The survey, comprising 35 questions, was administered in the local Indonesian language, which took approximately 30 min to complete. The study was conducted in accordance with the Checklist for Reporting Results of Internet E-Surveys (CHERRIES) to ensure the accuracy, validity, and reliability of the online survey methodology and data reporting (22).

### 2.2 Participants

Potential respondents from the GEMKAP study who opted in to participate in online surveys were recruited through an existing web-based panel via email invitations sent through the panel agencies’ mailing list. Eligible participants were adults between 21 and 60 years of age who provided consent to participate in the study. The upper age limit for this survey was set at 60 based on two factors: the higher prevalence of dengue among children and young adults in endemic countries, where prevention and management efforts have a greater impact, and the aim to reduce selection bias by limiting differences in digital literacy among participants. Individuals were excluded if they had participated in similar surveys within the past three months or were not personally responsible for their health. Participation was voluntary, and an incentive was provided upon completion of the full survey.

### 2.3 Electronic survey development

A survey was developed for the GEMKAP study by reviewing published dengue KAP studies to collect data on various aspects related to dengue. This included knowledge, attitudes, and practices regarding disease prevention, vaccination, and trusted stakeholders and preferences in communication channels for health information dissemination. Following the translation of the survey into Bahasa Indonesia, two cognitive qualitative interviews were conducted to refine, optimize and validate the survey for questionnaire robustness and local language comprehension. Data validation measures, including mandatory response constraints, Internet Protocol (IP) verification, identity validation, and digital fingerprinting, were implemented to ensure data quality and prevent duplicate responses. Engagement checks flagged irregular patterns, and a data cleaning program reviewed timestamps and response consistency to remove low-quality entries. Each participant could complete the survey only once, with duplicates excluded before analysis.

### 2.4 Covariates and outcomes

For the GEMKAP study, sociodemographic variables included gender, age, household size, ethnicity, religion, region of residence, level of education, and household income, along with other baseline characteristics including dengue experience, perceived risk, and vaccination history against dengue, COVID-19 and influenza. Primary outcomes focused on respondents’ willingness to vaccinate against dengue using a scale ranging from 0 to 10. A higher score indicated greater willingness to vaccinate, with 8 to 10 considered high willingness. A score of 4 to 7 was considered moderate willingness, and a score of 0 to 3 was considered low willingness. Secondary outcomes assessed overall Knowledge, Attitudes, and Practices toward dengue infection and symptoms, prevention methods, and vaccines. Each survey question was assigned to a subcategory of K, A or P to calculate a composite score for each subcategory (Supplementary Table 1). Composite scores for K, A and P were calculated and standardized to a scale of 0–100%, where scores of 80–100% were considered a “high” score, 50–79% a “moderate” score, and 49% or below a “low” score. These cut-offs were determined based on established methodologies used in prior KAP studies, including dengue-related research and other public health KAP assessments (21, 23).

### 2.5 Data analysis

Indonesia-specific data was extracted from the GEMKAP study, and further descriptive analysis was used to analyze sociodemographic variables, other baseline characteristic variables, as well as primary and secondary outcomes, using counts, percentages, and means. This approach provides an overview of the data, highlighting key trends, frequencies, and central tendencies. Subgroup analysis of the primary and secondary outcomes was conducted by covariates to ascertain the differences in willingness to vaccinate and levels of KAP across the covariate subgroups. These insights help identify potential patterns within specific populations, while the focus on descriptive analysis maintains clarity and transparency in the interpretation of the data. All analyses were performed using R, version 4.2.1.

### 2.6 Ethics and data confidentiality

The GEMKAP study received exemption status from the Pearl Institutional Review Board. Participants provided informed consent electronically, and data were handled anonymously in compliance with local privacy laws. Data were stored securely and accessed with permission.

The detailed methods of this study regarding study design, participant recruitment, electronic survey development (including validation and administration), data collection handling, study variables, data analysis and ethics and data confidentiality (including Institutional Review Board Statement), can be referenced from the original GEMKAP study (14).

## 3 Results

### 3.1 Response rate and socio-demographic characteristics of participants

For this Indonesian study, a total of 7,989 individuals accessed the screener questionnaire. Of these, 5,661 were disqualified, 585 dropped out, and 1,093 were terminated due to quota overfill. An additional 50 oversampled responses were excluded as the required 600 valid samples for analysis were already collected. The participation rate among those who accessed the screener questionnaire and the responses that were used for analysis was 7.5% (600/7,989). Table 1 displays the socio-demographic characteristics of the study participants. The distribution between males and females, as well as among different age groups, was relatively equal. The largest proportion of respondents (52%) resided in the Java region. Most respondents (61%) lived in moderate-sized households, consisting of 3–4 members, with a majority (61%) raising 1–2 children. A majority (68%) reported a high level of education, having completed tertiary education or higher. The predominant religion among respondents was Islam (81%). Among those surveyed, 38% reported a dengue diagnosis at some point in the past. This was based on respondents’ perceptions and not actual dengue infection serostatus.

**Table 1:** Socio-demographic characteristics of study respondents in Indonesia (*N* = 600).

Demographic	Sociodemographic	*N* (%)
Gender	Male	303 (50%)
	Female	297 (50%)
Age	21–30 years old	183 (30%)
	31–40 years old	168 (28%)
	41–50 years old	144 (24%)
	51–60 years old	105 (18%)
Household size	Live alone	24 (4%)
	1–2 members	58 (9%)
	3–4 members	362 (61%)
	5–6 members	139 (23%)
	>6 members	17 (3%)
Family household: children	No children	168 (28%)
	1–2 children	367 (61%)
	3–4 children	57 (10%)
	>4 children	8 (1%)
Religion	Christianity	83 (14%)
	Islam	488 (81%)
	Buddhism or Taoism	15 (3%)
	Others	14 (2%)
Education level	No formal education	1 (0%)
	Primary education	5 (1%)
	Secondary education	183 (31%)
	Tertiary education	384 (64%)
	Post-tertiary education	27 (4%)
Level of income	High (≥5,000,000 IDR)	87 (14%)
	Medium (1,500,000 – 4,999,999 IDR)	286 (48%)
	Low (<1,500,000 IDR)	227 (38%)
Prior dengue infection^1^	Yes	226 (38%)
	No	374 (62%)
Vaccinated against COVID-19	Yes	567 (95%)
	No	33 (5%)
Vaccinated against influenza	Yes	126 (21%)
	No	474 (79%)

^1^Based on respondents’ perception, regardless of their actual dengue infection serostatus.

### 3.2 Knowledge, attitudes and practices toward dengue and its prevention

The study assessed respondents’ Knowledge of dengue, Attitudes toward dengue transmission, diagnosis and risks, Practices of dengue vector control and mosquito bite prevention, and Knowledge of and Attitudes toward dengue vaccination and vaccine roll-out. The study results revealed encouraging levels of Attitude (65%), followed by Practice (56%), with room for improvement in Knowledge levels (46%) in Indonesia.

**Figure 1:**
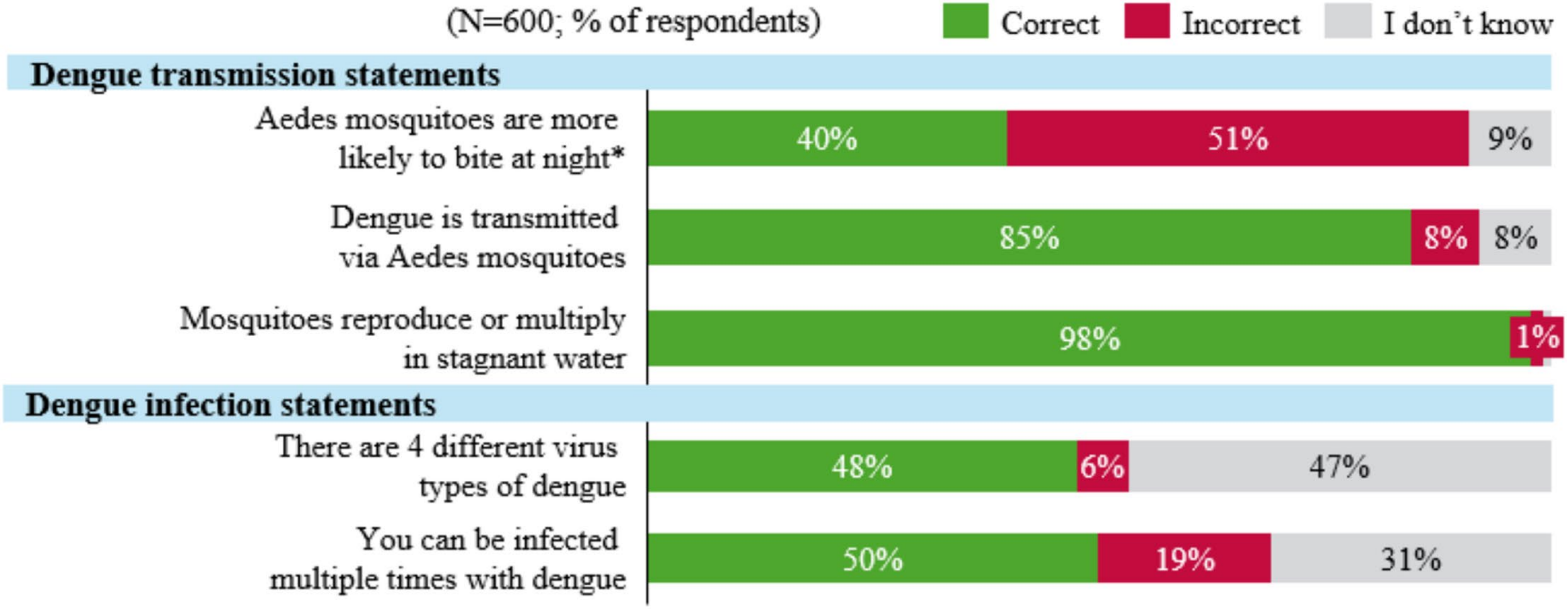
Knowledge regarding dengue transmission and infection in Indonesia. ^*^Represents an incorrect statement.

Figure 1 summarizes the knowledge levels and gaps regarding dengue transmission and infection. The majority were aware that dengue is transmitted via the *Aedes* mosquito (85%) and that mosquitoes reproduce in stagnant water (98%). Most respondents also recognized common dengue symptoms such as body aches (72%) and body chills (61%). However, there were knowledge gaps on dengue infection modes, with low awareness of the four dengue serotypes (48%), and the possibility of multiple infections from different serotypes (50%).

Dengue was perceived to be a severe disease by 67% of respondents, and most understood the potential for hospitalization due to the infection (79%). However, awareness of additional clinic visits (46%), the risk of reinfection (33%), increased severity due to reinfection (19%), and reduced quality of life post-infection (28%) was less widespread. Regarding the financial impact of contracting dengue, the majority recognized that there may be additional hospitalization costs (54%) not covered by national health insurance or *Jaminan Kesehatan Nasional (JKN)*, such as fees for upgraded hospital rooms and specialist consultations. Additionally, 60% of respondents were aware that dengue infection may lead to absenteeism from school or work, but only 23% understood the potential additional costs associated with hiring support, such as caregivers, for mild to severe dengue cases.

Lastly, there were knowledge gaps on the availability of dengue treatments and vaccines. The majority of respondents believed that there is a cure for dengue (69%), while there is currently no available specific treatment for dengue. Additionally, most respondents were uncertain or unaware of the availability of dengue vaccines (60%) in Indonesia or globally.

### 3.3 Attitudes and perceptions toward various dengue prevention methods

**Figure 2:**
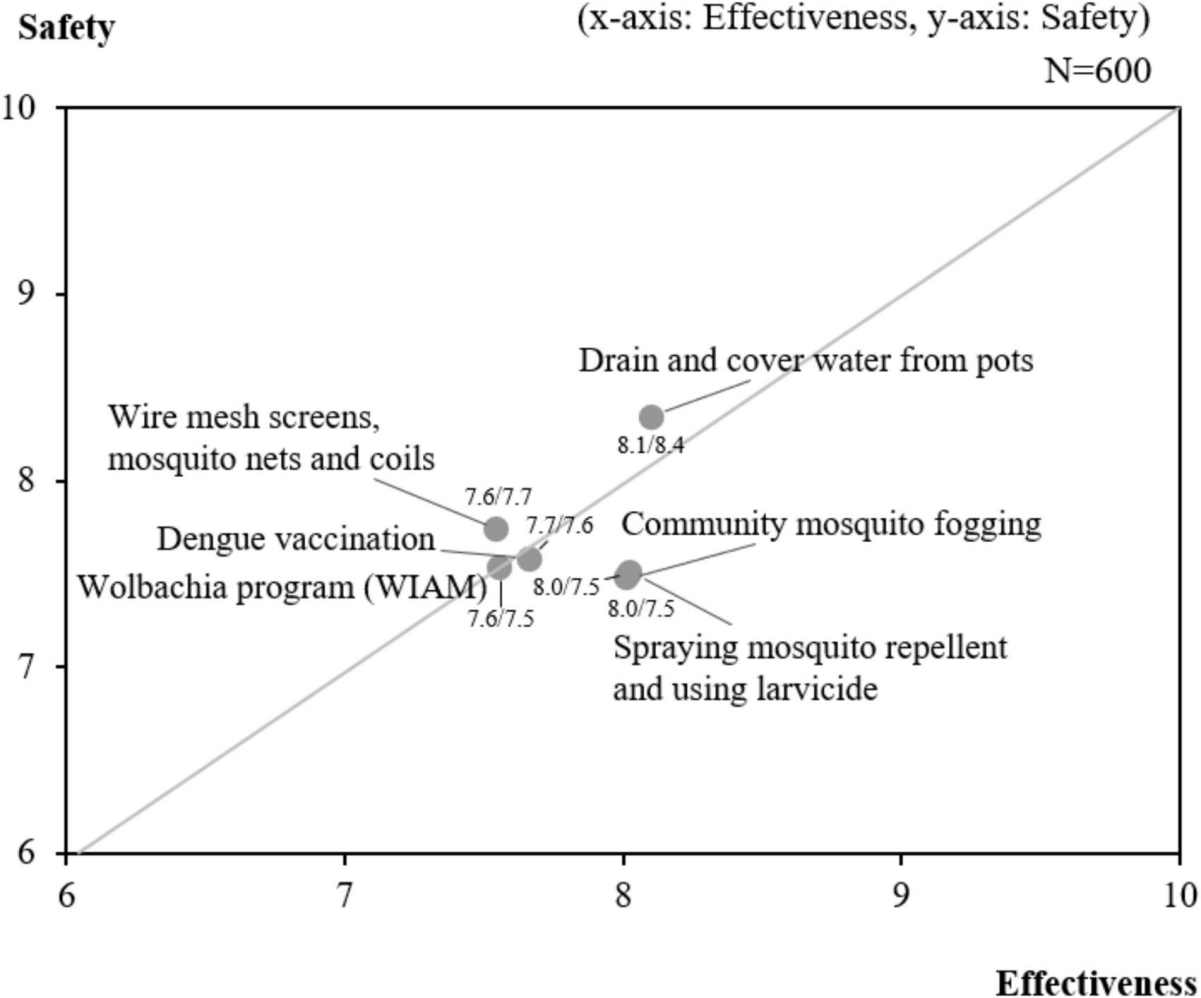
Attitudes on the safety and effectiveness of dengue prevention methods.

Figure 2 presents a summary of attitudes and perceptions regarding various dengue prevention methods, including vector control techniques and dengue vaccination. Overall, most prevention methods evaluated were perceived as generally safe (average score of 7.7, out of a scale of 0–10) and effective (average score of 7.8). Among all vector control methods, draining and covering water containers was perceived to be the safest (8.4), followed by using wire mesh screens, mosquito nets and coils (7.7) and dengue vaccines (7.6). Regarding effectiveness, draining and covering water containers (8.1), community mosquito fogging (8.0) and spraying mosquito repellent (8.0) were perceived to be the most effective.

### 3.4 Dengue prevention practices

**Figure 3:**
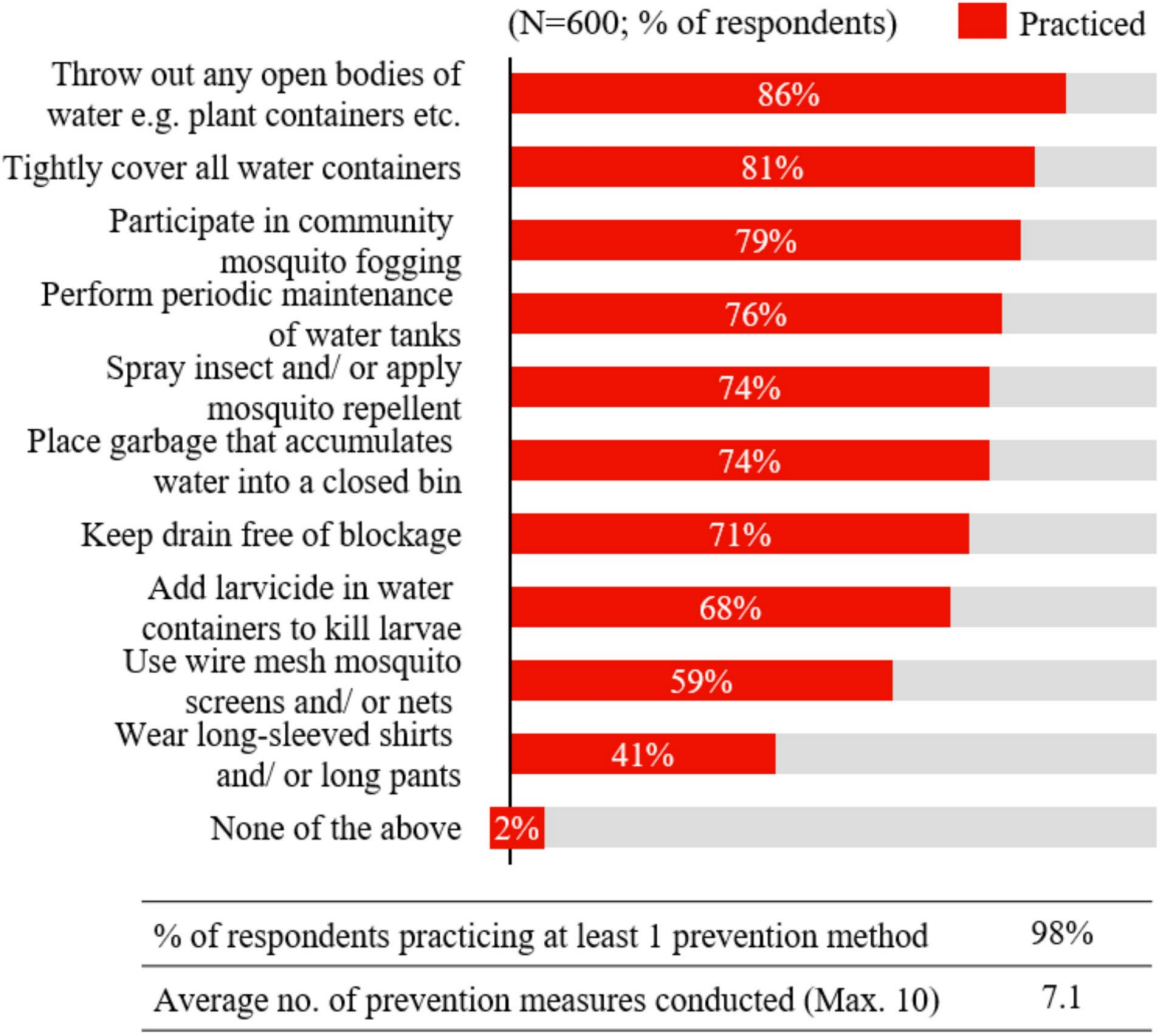
Prevention measures practiced.

**Figure 4:**
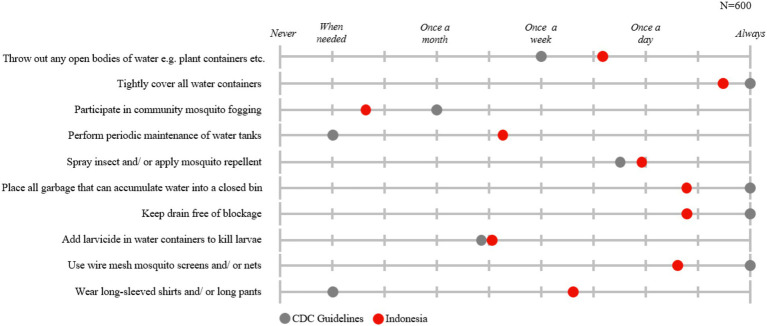
Frequency of prevention measures practiced.

Regarding the practice of dengue prevention, Figure 3 illustrates that nearly all Indonesian respondents (98%) reported practicing at least one (out of 10) prevention measure. On average, respondents engaged in 7 out of the 10 prevention methods evaluated in the study. The most practiced methods were disposing of open bodies of water (86%) and tightly covering all water containers (81%). These prevention measures are practiced as often or more frequently than recommended by CDC guidelines (24), as shown in Figure 4. Respondents reported disposing of open bodies of water several times a week, exceeding the recommended frequency of once a week, and almost always covering water containers, which aligns with the guidelines. Conversely, the least practiced prevention methods were wearing long sleeves or long pants (41%) and using wire mesh mosquito screens and/or nets (59%). However, when adopted, respondents reported wearing long-sleeves and long pants several times a week—more frequently than the recommended ‘only when needed’— and using mosquito screens and nets consistently.

### 3.5 Attitudes toward dengue vaccination

**Figure 5:**
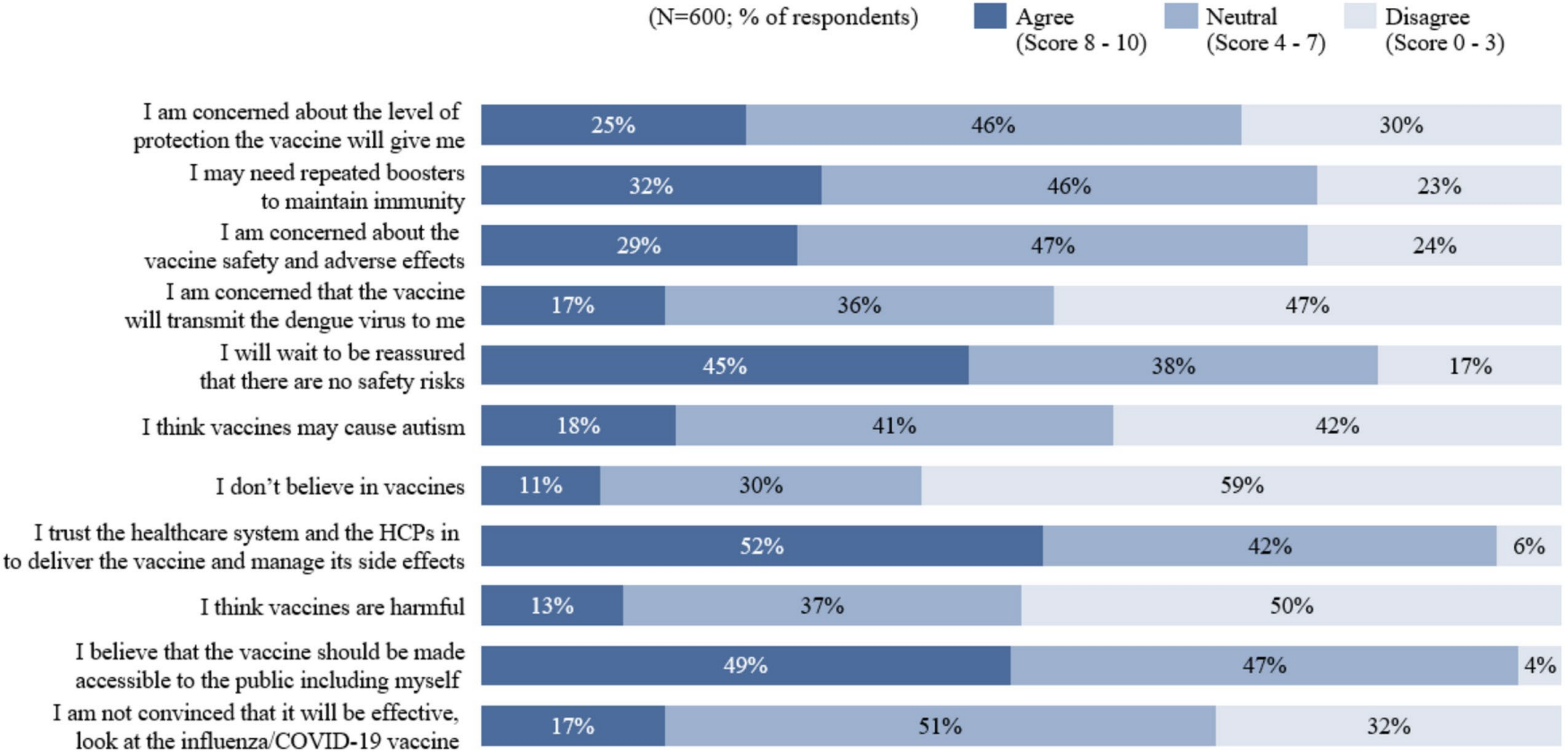
Attitude regarding dengue vaccines in Indonesia.

Figure 5 presents a summary of attitudes regarding dengue vaccines in Indonesia. The results showed that the majority of respondents had a positive outlook toward dengue vaccination. Out of 600 respondents, only 11% did not believe in dengue vaccines, 17% were unconvinced of their effectiveness, and 13% perceived them as harmful. These sentiments are also echoed by an overall positive attitude toward general vaccination, with 74% of respondents acknowledging the importance of vaccines in preventing infectious diseases, and 65% identifying themselves as pro-vaccination (Supplementary Figure 1). However, nearly half (45%) of respondents agreed that they would wait to be reassured that there were no safety risks before taking a dengue vaccine, even if approved by global or local health authorities. Additionally, 29% agreed that they were concerned about the vaccines’ safety and adverse effects, and 25% were concerned about the level of protection it offers.

### 3.6 Willingness to vaccinate against dengue disease and acceptance of a hypothetical dengue vaccine

Approximately half (51%) of Indonesian participants expressed willingness (scoring 8 to 10) to be vaccinated against dengue. The willingness level increased to 58% when recommended by a physician. Among those willing to be vaccinated, the top three reasons were the desire for protection against dengue (30%), the intention to boost one’s immunity (19%), and the belief that vaccines are important for overall health protection (15%). Conversely, factors contributing to a low willingness to vaccinate included fear of side effects (18%), fear of needles (12%), and the perception that vaccines are not effective due to limited evidence (9%).

Table 2 provides an overview of individual willingness to vaccinate against dengue across various socio-demographic sub-groups. Sub-groups with higher willingness to vaccinate included those with 1–2 children, who were more likely to vaccinate (56%) compared to those without children (41%). Additionally, respondents vaccinated against COVID-19 expressed a higher willingness to vaccinate against dengue (53%) compared to those who were not vaccinated (15%). Moreover, respondents with a highly positive opinion of vaccines, regarding them as ‘very useful’, showed greater willingness to vaccinate (69%) than those who perceived them as only ‘somewhat useful’ (14%). Lastly, respondents with a ‘very high risk’ perception of dengue had a higher willingness to vaccinate (64%) compared to those who considered dengue as ‘medium risk’ (29%).

**Table 2:** Individual willingness to vaccinate against dengue in Indonesia by socio-demographic sub-groups (*N* = 600).

Demographic	Sociodemographic	Base	Mean	Standard Deviation	Willingness to vaccinate against dengue (*N*, %)		
					High willingness	Moderate willingness	Low willingness
Gender	Male	303	7.4	2.1	164 (54%)	121 (40%)	18 (6%)
	Female	297	7.2	2.1	144 (49%)	137 (46%)	16 (5%)
Age	21–30 years old	183	6.8	2.2	77 (42%)	92 (50%)	15 (8%)
	31–40 years old	168	7.7	2.0	101 (60%)	59 (35%)	8 (5%)
	41–50 years old	144	7.5	2.1	82 (57%)	55 (38%)	7 (5%)
	51–60 years old	105	7.3	1.9	47 (45%)	54 (51%)	4 (4%)
Family household: children	No children	168	6.8	2.3	69 (41%)	86 (51%)	13 (8%)
	1–2 children	367	7.5	2.0	206 (56%)	147 (40%)	15 (4%)
	3–4 children	57	7.1	2.3	28 (49%)	24 (42%)	5 (9%)
	>4 children	8	7.8	1.4	5 (63%)	3 (37%)	-
Religion	Christianity	83	7.7	2.1	51 (61%)	29 (35%)	3 (4%)
	Islam	488	7.2	2.1	244 (50%)	215 (44%)	29 (6%)
	Buddhism or Taoism	15	6.9	2.1	5 (33%)	10 (67%)	-
	Others	14	7.5	1.6	7 (50%)	7 (50%)	-
Education level	No formal education	1	5.0	0.0	-	1 (100%)	-
	Primary education	5	5.4	1.1	-	5 (100%)	-
	Secondary education	183	6.9	2.4	86 (47%)	79 (43%)	18 (10%)
	Tertiary education	380	7.5	1.9	204 (54%)	165 (43%)	12 (3%)
	Post-tertiary education	27	7.2	2.5	16 (59%)	8 (30%)	3 (11%)
Level of Income	High	87	7.4	1.9	44 (51%)	40 (46%)	3 (3%)
	Medium	286	7.4	1.9	150 (53%)	127 (44%)	9 (3%)
	Low	227	7.1	2.4	113 (50%)	92 (41%)	22 (9%)
Prior dengue infection	Yes	226	7.6	1.9	131 (58%)	87 (39%)	8 (3%)
	No	374	7.1	2.2	176 (47%)	172 (46%)	26 (7%)
Vaccinated against COVID-19	Yes	567	7.4	1.9	301 (53%)	244 (43%)	23 (4%)
	No	33	4.8	3.0	5 (15%)	18 (55%)	10 (30%)
Vaccinated against influenza	Yes	127	7.6	1.8	76 (60%)	47 (37%)	5 (3%)
	No	474	7.2	2.2	232 (49%)	213 (45%)	28 (6%)
Level of perceived risk	Very high risk	244	7.9	2.0	155 (64%)	80 (33%)	9 (3%)
	High risk	220	7.3	1.7	110 (50%)	101 (46%)	9 (4%)
	Medium risk	101	6.5	2.0	29 (29%)	64 (63%)	8 (8%)
	Low risk	21	6.5	2.8	10 (48%)	8 (38%)	3 (14%)
	Very low risk	8	5.3	3.4	2 (24%)	3 (38%)	3 (38%)
	No risk	6	3.7	4.1	1 (17%)	2 (33%)	3 (50%)
Opinion toward vaccines	Very useful	305	8.1	1.8	209 (69%)	90 (30%)	6 (1%)
	Useful	210	7.0	1.6	85 (41%)	116 (55%)	9 (4%)
	Somewhat useful	63	5.7	2.0	9 (14%)	45 (71%)	9 (15%)
	Slightly useful	13	5.5	2.7	3 (23%)	8 (62%)	2 (15%)
	Not useful	4	0.5	0.6	-	-	4 (100%)
	Not useful at all	5	2.4	4.3	1 (20%)	-	4 (80%)

### 3.7 Preferred methods and stakeholders for seeking health-related information

In Indonesia, search engines (88%) and social media channels (85%) were identified as the primary preferred methods for seeking health information (Supplementary Figure 2). Healthcare professionals (HCPs) were considered the most trusted stakeholders for communicating health-related information, with doctors being the most trusted (94%), followed by nurses/paramedics (55%), and pharmacists (34%) (Supplementary Figure 3). Additionally, government bodies (49%) were also perceived as one of the trusted sources of health information.

### 3.8 Preference for a multi-pronged dengue management program

Regarding the population’s preference for the three approaches (vector control, vaccination and education) to dengue management, a comprehensive strategy combining vaccination, education and vector control programs was the most preferred, with 42% of the Indonesian population supporting its implementation. This was followed by a preference for a vaccination program and education program (37%), and lastly, a vaccination and vector control program (15%). Further details of the results can be found in Supplementary Figure 4.

## 4 Discussion

### 4.1 Knowledge, attitude and practice (KAP) levels and gaps in dengue disease and prevention

The GEMKAP study provides a comprehensive description of Knowledge, Attitudes and Practices (KAP) regarding dengue disease and prevention in Indonesia. These findings are important to inform and contribute to dengue management programs to reduce Indonesia’s dengue burden.

The study revealed a gap in dengue awareness among Indonesians, consistent with findings from other South-East Asian countries (Singapore and Malaysia) evaluated in the GEMKAP study, indicating an opportunity for improvement in Knowledge levels (46%) (14). Respondents demonstrated high awareness of specific aspects of transmission, such as mosquito reproduction in stagnant water and dengue transmission via the *Aedes* mosquito. Additionally, awareness of mosquito behavior, such as their increased activity and biting frequency in hot or humid weather, was relatively high. However, knowledge was lower regarding other critical aspects such as the existence of multiple dengue virus serotypes, the risk of secondary infections and mosquitoes’ heightened activity during daylight hours. This finding aligns with a previous study in Yogyakarta, Indonesia, which revealed that less than half of the respondents were aware that mosquitoes are primarily active during the day (13).

Additionally, although most respondents from the study recognized common symptoms of dengue, such as fever and chills, these symptoms are also present in other febrile diseases. This potential lack of differentiation in dengue symptoms compared to other febrile diseases may result in delays in seeking appropriate healthcare, as dengue may not be immediately considered. Consequently, this may lead to delayed diagnosis and management, increasing the risk of severe manifestations of the disease, such as dengue hemorrhagic fever and dengue shock syndrome. These severe forms of dengue often require long-term or intensive care, leading to significant health and economic impacts (25, 26).

Furthermore, findings from the GEMKAP study indicate that respondents may lack awareness of the broader consequences of dengue infection. While there was awareness of the direct impact of dengue contraction, such as hospitalization, the long-term implications, including increased susceptibility to severe disease with re-infection and reduced quality of life post-infection, appear less understood. Additionally, the indirect financial impacts of severe dengue, such as the potential additional hospitalization-related costs, productivity losses and caregiving expenses, were also underappreciated. This finding aligns with studies conducted across Asia (26, 27). This limited awareness may contribute to a lack of urgency in adopting consistent preventive measures.

Attitudes toward dengue prevention methods were generally positive in Indonesia. On average, respondents viewed most vector control methods as generally safe and effective. However, there were some variations in perceived levels of safety and effectiveness between the different methods. For instance, draining open bodies of water and community mosquito fogging were considered effective, while fogging was seen as less safe. In contrast, wire mesh screens, mosquito nets and coils were perceived as generally safe but less effective. When compared to more established vector control methods, perceptions of newer prevention methods, such as *Wolbachia* and vaccinations, did not differ significantly in terms of safety and effectiveness.

Practice levels for dengue prevention were notably high in Indonesia, with nearly all respondents practicing at least one prevention measure and an average of seven out of ten measures adopted per respondent. This suggests that the importance of prevention has been generally well-understood and emphasized. However, certain methods, such as wearing long sleeves and using wire mesh mosquito screens, were less commonly practiced, highlighting areas for improvement. Overall, Indonesians frequently adhered to or exceeded CDC guidelines, reflecting a strong grasp of the significance of preventative measures (24). Furthermore, a study in Indonesia found that higher education levels were correlated with increased engagement in dengue prevention practices, consistent with the GEMKAP study’s Indonesian results. This highlights the potential need to tailor communication strategies according to education levels (21).

More specifically, when examining KAP results on dengue vaccines, it was found that the awareness of the availability of dengue vaccines was low. This is consistent with an Indonesian study, which found that awareness of the new rotavirus was low as it was not on the National Immunization program (28). Moreover, concerns about potential side effects and doubts regarding vaccine effectiveness were identified as primary reasons for vaccine hesitancy. Similarly, with COVID-19 vaccines, hesitancy was driven by concerns such as safety and side effects, alongside a lack of trust, misinformation, and insufficient information (29).

Addressing the identified KAP gaps is crucial for improving dengue prevention efforts in Indonesia. While there is considerable awareness of dengue transmission methods and generally positive attitudes toward existing prevention methods, gaps remain in the awareness of implications due to dengue contraction and the availability, effectiveness and safety of newer prevention strategies such as vaccines. This study serves as a baseline for future research, allowing for subsequent assessments to help translate awareness into effective practice and ensure consistent use of all preventive measures (30).

### 4.2 Addressing the knowledge, attitude and practice (KAP) gaps via an integrated dengue prevention and control approach

To effectively address the identified KAP gaps, an integrated dengue management strategy comprising vector control, vaccination and education is essential. In this approach, vector control and vaccination programs can be reinforced by education programs that address the pre-identified knowledge and attitude gaps on the safety and effectiveness of dengue prevention methods. This could thereby foster positive attitudes and encourage participation and adherence at both the community and individual levels.

This comprehensive integrated approach, which includes vector control, vaccination, community education and engagement, and proper case management, aligns with the World Health Organization’s (WHO) recommendation on dengue management (1). To further affirm the importance of an integrated approach, a previous study in Indonesia illustrated that no single intervention is sufficient to reduce the burden of dengue effectively (13). With this aim, Indonesia’s Ministry of Health adopted a similar approach through the National Dengue Control Strategy 2021–2025 (‘Strategi Nasional (STRANAS) Penanggulangan Dengue 2021–2025’), which incorporates elements of vector control, vaccination and education (31). As part of STRANAS, the community-based program 3 M Plus which translates to “Menutup” (cover water containers), “Menguras” (drain open water bodies) and “Mendaur ulang” (recycle unused containers) is a key component comprising multiple vector control measures and the Plus refers to additional measures to prevent mosquito bites (13). These programs are bolstered by public health campaigns, such as disseminating health education through mass media (13). Alongside these efforts, *Wolbachia* and vaccination programs have been rolled out as an additional measure to strengthen vector control and dengue prevention.

To enhance existing dengue programs and address the identified KAP gaps in Indonesia, it is essential to define the key focus areas and develop clear messages for vector control, vaccination, and education. These messages should be targeted, easily digestible, and reliable to address misconceptions, provide evidence-based information, and build public trust, particularly in newer prevention methods. The following sections provide examples of focus areas and key messages for vector control, vaccination and education programs.

#### 4.2.1 Vector control

Based on the GEMKAP results, Indonesian respondents are already actively engaged in dengue prevention measures. For vector control education, it is essential to emphasize the importance of consistent practices and understanding the benefits of managing these measures independently. For example, regular reminders should be sent to individuals to cover up open water bodies and share disease surveillance data to highlight any decrease in cases. Making these practices routine and a natural part of daily behavior can help improve the impact of vector control. This focus is particularly critical in highly endemic regions or areas with dengue hotspots.

Newer dengue vector control methods, such as *Wolbachia,* are also being implemented in Indonesia, which currently covers six cities – Yogyakarta, Semarang, Kupang, Bandung, West Jakarta and Bontang (16). It will be important to educate the community about the process and benefits of *Wolbachia* while addressing concerns about its perception of low safety and effectiveness compared to traditional vector control methods. For instance, highlighting *Wolbachia’s* long-term advantages, such as reducing reliance on insecticides, combating mosquito resistance, and being environmentally friendly, can enhance its appeal (32). This strategy complements existing prevention efforts as a safe and sustainable tool that will foster greater community acceptance.

#### 4.2.2 Vaccination

Next, dengue vaccination is another vital component of dengue prevention. However, gaps in knowledge about vaccine availability and concerns about potential side effects may contribute to vaccine hesitancy. To address these issues, it is crucial to enhance public awareness of the availability and benefits of dengue vaccines while demonstrating their safety and effectiveness. Building public confidence and trust is key to improving vaccine acceptance (33). Concerning the education component of vaccines, the messaging can focus on the availability of dengue vaccines, safety and effectiveness and risk of side effects.

Firstly, concerning the availability of vaccines, as of the GEMKAP study (October 2022), two dengue vaccines, CYD-TDV and TAK-003, have been approved by the Indonesian regulatory body, Badan Pengawas Obat dan Makanan (BPOM). CYD-TDV was approved in August 2016 for 9–16 year olds (34), with the requirement for pre-vaccination screening announced in February 2018. More recently, TAK-003 was approved in August 2022 for individuals aged 6–45 years, representing a broader age range inclusion in vaccination efforts (35, 36). However, it is worth noting that while both vaccines are approved, neither has been distributed under a nationally supported program, highlighting opportunities to improve access and delivery.

Secondly, to address safety concerns, it is important to communicate that both vaccines (CYD-TDV and TAK-003) have been recommended by the WHO and the Strategic Advisory Group of Experts on Immunization (SAGE) for use in public settings in countries with high dengue transmission. This recommendation aims to reduce dengue cases and hospitalizations and maximize the public health impact (37). At the national level, both CYD-TDV and TAK-003 dengue vaccines are recommended by the Indonesian Paediatricians Society (Ikatan Dokter Anak Indonesia – IDAI) (38). Additionally, TAK-003 has been recommended by the Indonesian Society of Internal Medicine (Perhimpunan Dokter Spesialis Penyakit Dalam Indonesia – PAPDI) and Indonesian Occupational Medicine Association (Perhimpunan Spesialis Kedokteran Okupasi Indonesia – PERDOKI) (18, 19).

Lastly, increasing awareness of the dengue vaccine may emphasize its role as an important tool for self-protection, complementing ongoing community efforts. It will be important to highlight the vaccine’s ability to reduce severe dengue manifestations and its potential to alleviate the clinical and financial burdens related to hospitalization, caregiving expenses, and productivity losses (15). Communicating advancements in vaccine development with updated safety and efficacy evidence will help dispel misconceptions.

A public dengue vaccination program was initiated by the East Kalimantan regional government at the end of 2023 (20). Although still in the early stages, these initiatives demonstrate the potential to adopt newer strategies and the opportunities to expand the scale of these programs. Expanding existing pilot vaccination programs to additional highly endemic areas in Indonesia will be important for improving access and optimizing coverage. Additionally, a public campaign will be crucial to tackle current KAP gaps related to vaccine accessibility, safety, and effectiveness, thereby mitigating vaccine hesitancy (39).

#### 4.2.3 Education

To optimize the implementation of vector control and vaccination measures, education programs should be used to address the identified knowledge gaps, correct misconceptions, and improve attitudes toward dengue disease and its prevention. Effective education involves implementing comprehensive, targeted campaigns to enhance understanding of dengue transmission, infection, symptoms, and preventive measures, including the risks of multiple virus serotypes and reinfection. Community workshops, school programs, and media campaigns can provide clear, accessible information to demystify dengue and encourage timely healthcare-seeking behavior (40–42).

Once targeted messaging is developed, selecting the most effective communication methods becomes crucial, as strong communication and health education are foundational for garnering support and adoption of new health initiatives and preventive measures (43). To reach a broad audience, dengue-related communication should be disseminated through digital outlets and traditional channels such as newspapers and television (44). Recent research in Indonesia has similarly shown that digital media, including websites, social media and mobile apps, are widely used to convey health information on topics such as disease prevention (45, 46).

By integrating education, vector control, and vaccination into a cohesive strategy and embracing innovative tools, we can address the identified KAP gaps comprehensively. This integrated approach may enhance public understanding, improve preventive practices, and increase vaccine coverage, ultimately leading to a more effective dengue management program and a reduction in the disease burden.

### 4.3 Collaboration among key stakeholders for an effective integrated dengue management plan

To ensure the successful design and implementation of an integrated dengue management program, strong collaboration among various stakeholders is crucial (47, 48). This has also been emphasized as a critical factor in successful dengue control programs across several Southeast Asian countries (49, 50). Key stakeholders in this process include government ministries, technical experts, HCPs and medical societies, community leaders and community healthcare workers (47). Clearly defining roles, responsibilities, and objectives is essential for synergizing efforts and maximizing the program’s impact (47).

Effective dengue management requires the collaboration of various stakeholders, each contributing to prevention efforts (47). Government bodies play a pivotal role by mobilizing resources to support the nationwide rollout of the vaccine, setting guidelines, and enacting national policies. Academics and clinical experts provide technical insights for evidence-based programs, while community leaders and health workers encourage public participation in prevention activities. Moreover, HCPs affiliated with medical associations contribute valuable insights into dengue management strategies, drawing on their clinical expertise and frontline experiences. Despite respective responsibilities, each stakeholder shares a collective role in enhancing dengue prevention through coordinated efforts. For instance, government agencies and community health workers can jointly communicate essential information about dengue prevention methods, such as safety and efficacy information, while medical associations and policymakers work together to integrate the latest scientific data into clinical guidelines.

Healthcare professionals play a uniquely trusted role in dengue prevention. The GEMKAP study highlights that HCPs are considered the most reliable source of health information by Indonesian respondents. Their influence is crucial in conveying health information about infectious diseases and vaccinations (51–53). To leverage this role, it is important to tailor key messages that address the knowledge, attitudes and practice gaps identified in the GEMKAP study (14). This approach should consider cultural sensitivities and target diverse sociodemographic groups to optimize its impact (54). HCPs can significantly contribute to dispelling misconceptions, and providing accurate information to educate the public about vaccine safety and efficacy, thereby reducing misconceptions about dengue and vaccine hesitancy (52). Additionally, they motivate and reassure individuals, encouraging vaccinations (55). Through these efforts, HCPs support informed decision-making and help build public confidence in participating in various dengue prevention activities.

### 4.4 Strengths and limitations

The GEMKAP study offers valuable and comprehensive insights into knowledge, attitudes and practices related to dengue, prevention methods, vaccines and health communication strategies. By utilising a large cross-sectional sample, it achieved broad generalizability across Indonesia while capturing a wide range of socio-demographic factors for a nuanced understanding of differences in knowledge, attitudes and practices.

However, there are several limitations to the GEMKAP Indonesia study. First, self-reported data may introduce recall and social desirability bias, as respondents may inaccurately recall past dengue exposure or overreport prevention behaviors to align with societal expectations. To minimize the social desirability bias, the survey was self-administered and anonymous, reducing pressure on respondents to provide socially desirable answers. Future studies could further minimize bias by incorporating objective behavioral measures, such as direct observation or community-level data. Second, while quotas were set for gender, age, income and region to ensure the representativeness of the adult population in Indonesia, potential sample bias may still exist as other sociodemographic variables such as education level were not controlled for. Third, the web-based survey may have favored participants with higher literacy and internet access, introducing selection bias to those who are already interested in health issues. This method also prioritizes responses from those with internet access and those living in extremely rural areas. Future studies could address these by controlling for education and expanding the scale of offline data collection. Fourth, the study did not compare results between respondents and non-respondents, which may limit the generalizability of the study’s findings. Additionally, the absence of multivariate regression analysis means that the study did not account for potential confounding factors, limiting our ability to identify independent predictors of knowledge, attitudes, and practices. Future research could build upon these findings by incorporating multivariate regression techniques, such as logistic regression, to provide deeper analysis and control for confounding variables. Lastly, the data collection period for the GEMKAP study was from September to October 2022, which may not fully capture shifts in dengue and vaccine sentiment that may have occurred subsequently. Biennial replication of the study could better capture these trends over time.

## 5 Conclusion

Indonesia’s GEMKAP study results showed positive attitude levels (65%) and moderate practice levels (56%) toward dengue but revealed significant gaps in knowledge (46%). While many Indonesians actively take preventive measures, there are awareness gaps regarding dengue transmission, severity, and the consequences of infection, as well as the safety and effectiveness of prevention methods like *Wolbachia* and vaccination. To address these gaps, an integrated program focused on vector control, vaccination, and education is necessary, aligning with respondents’ preference for such an approach. Furthermore, most Indonesians view healthcare professionals as trusted sources of health information. Therefore, collaborative efforts among diverse stakeholders are essential for the successful implementation of dengue management strategies.

## Data Availability

The original contributions presented in the study are included in the article/Supplementary material, further inquiries can be directed to the corresponding author.

## References

[1] Dengue-Global situation. (2024). Available online at: https://www.who.int/emergencies/disease-outbreak-news/item/2023-DON498 (Accessed March 7, 2024).

[2] Utama IMS Lukman N Sukmawati DD Alisjahbana B Alam A Murniati D. Dengue viral infection in Indonesia: epidemiology, diagnostic challenges, and mutations from an observational cohort study. PLoS Negl Trop Dis. (2019) 13:e0007785. doi: 10.1371/journal.pntd.0007785, PMID: 31634352 PMC6822776

[3] World Mosquito Program. (2024). World mosquito program in Indonesia. Available online at: https://www.worldmosquitoprogram.org/en/global-progress/indonesia (Accessed April 30, 2024).

[4] Dhewantara PW Marina R Puspita T Ariati Y Purwanto E Hananto M. Spatial and temporal variation of dengue incidence in the island of Bali, Indonesia: an ecological study. Travel Med Infect Dis. (2019) 32:101437. doi: 10.1016/j.tmaid.2019.06.008, PMID: 31362115

[5] Sasmono RT Kalalo LP Trismiasih S Denis D Yohan B Hayati RF. Multiple introductions of dengue virus strains contribute to dengue outbreaks in East Kalimantan, Indonesia, in 2015–2016. Virol J. (2019) 16:93. doi: 10.1186/s12985-019-1202-0, PMID: 31345242 PMC6659258

[6] Indriani C Ahmad RA Wiratama BS Arguni E Supriyati E Sasmono RT. Baseline characterization of dengue epidemiology in Yogyakarta City, Indonesia, before a randomized controlled trial of Wolbachia for Arboviral disease control. Am J Trop Med Hyg. (2018) 99:1299–307. doi: 10.4269/ajtmh.18-0315, PMID: 30226138 PMC6221224

[7] Astuti EP Dhewantara PW Prasetyowati H Ipa M Herawati C Hendrayana K. Paediatric dengue infection in Cirebon, Indonesia: a temporal and spatial analysis of notified dengue incidence to inform surveillance. Parasit Vectors. (2019) 12:186. doi: 10.1186/s13071-019-3446-3, PMID: 31036062 PMC6489314

[8] Fauzi IS Nuraini N Ayu RWS Lestari BW. Temporal trend and spatial clustering of the dengue fever prevalence in West Java, Indonesia. Heliyon. (2022) 8:e10350. doi: 10.1016/j.heliyon.2022.e10350, PMID: 36061000 PMC9433680

[9] Kementerian Kesehatan Republik Indonesia. Laporan Tahunan 2022: Demam Berdarah Dengue. Jakarta: Direktorat Pencegahan dan Pengendalian Penyakit Menular (2023). 17:20.

[10] Kosasih H Alisjahbana B Nurhayati d MQ Rudiman IF Widjaja S Antonjaya U. The epidemiology, virology and clinical findings of dengue virus infections in a cohort of Indonesian adults in Western Java. PLoS Negl Trop Dis. (2016) 10:e0004390. doi: 10.1371/journal.pntd.0004390, PMID: 26872216 PMC4752237

[11] Dengue in Indonesia. International Federation of red Cross and red Crescent Societies. Available online at: https://www.ifrc.org/sites/default/files/2022-10/Dengue-Indonesia-Prevent-Epidemics.pdf (Accessed February 23, 2024).

[12] Wilastonegoro NN Kharisma DD Laksono IS Halasa-Rappel YA Brady OJ Shepard DS. Cost of dengue illness in Indonesia across hospital, ambulatory, and not medically attended settings. Am J Trop Med Hyg. (2020) 103:2029–39. doi: 10.4269/ajtmh.19-0855, PMID: 32901596 PMC7646801

[13] Sulistyawati S Dwi Astuti F Rahmah Umniyati S Tunggul Satoto TB Lazuardi L Nilsson M. Dengue vector control through community empowerment: lessons learned from a community-based study in Yogyakarta, Indonesia. Int J Environ Res Public Health. (2019) 16:1013. doi: 10.3390/ijerph16061013, PMID: 30897770 PMC6466136

[14] Shafie AA Moreira ED Di Pasquale A Demuth D Yin JYS. Knowledge, attitudes and practices toward dengue fever, vector control, and vaccine acceptance among the general population in countries from Latin America and Asia Pacific: a cross-sectional study (GEMKAP). Vaccine. (2023) 11:575. doi: 10.3390/vaccines11030575, PMID: 36992159 PMC10058638

[15] Suwantika AA Kautsar AP Supadmi W Zakiyah N Abdulah R Ali M. Cost-effectiveness of dengue vaccination in Indonesia: considering integrated programs with Wolbachia-infected mosquitos and health education. Int J Environ Res Public Health. (2020) 17:4217. doi: 10.3390/ijerph17124217, PMID: 32545688 PMC7345186

[16] Wolbachia: A natural solution to fighting dengue in Bali. (2024). World mosquito program. Available from: https://www.worldmosquitoprogram.org/en/news-stories/stories/wolbachia-natural-solution-fighting-dengue-bali (Accessed June 6, 2024).

[17] Zeng W Halasa-Rappel YA Baurin N Coudeville L Shepard DS. Cost-effectiveness of dengue vaccination in ten endemic countries. Vaccine. (2018) 36:413–20. doi: 10.1016/j.vaccine.2017.11.064, PMID: 29229427

[18] Jadwal Imunisasi Dewasa. PAPDI. (2024). Available online at: https://satgasimunisasipapdi.com/jadwal-imunisasi-dewasa/ (Accessed April 17, 2024).

[19] Sulistomo A Zamsiar N Jasmine R Puspitasari A Hutapea R. Panduan Imunisasi Untuk Perlindungan Dan Upaya Peningkatan Produktifitas Pekerja. (2024). Available online at: https://event.perdoki.or.id/products/show/9b6cf802-cf59-4f9e-a7b0-009c4ba89039 (Accessed March 18, 2025).

[20] Dinas Kesehatan Provinsi Kaltim. (2025). Memperingati Bulan K3 Pertamina RU V Gelar Vaksinasi DBD Untuk Karyawan, Kadinkes Apresiasi. Available online at: https://dinkes.kaltimprov.go.id/single-berita/112 (Accessed March 18, 2024).

[21] Harapan H Rajamoorthy Y Anwar S Bustamam A Radiansyah A Angraini P. Knowledge, attitude, and practice regarding dengue virus infection among inhabitants of Aceh, Indonesia: a cross-sectional study. BMC Infect Dis. (2018) 18:96. doi: 10.1186/s12879-018-3006-z, PMID: 29486714 PMC5830327

[22] Eysenbach G. Improving the quality of web surveys: the checklist for reporting results of internet E-surveys (CHERRIES). J Med Internet Res. (2004) 6:e34. doi: 10.2196/jmir.6.3.e34, PMID: 15471760 PMC1550605

[23] Al-Zalfawi SM Rabbani SI Asdaq SMB Alamri AS Alsanie WF Alhomrani M. Public knowledge, attitude, and perception towards COVID-19 vaccination in Saudi Arabia. Int J Environ Res Public Health. (2021) 18:10081. doi: 10.3390/ijerph181910081, PMID: 34639382 PMC8508088

[24] CDC. Mosquitoes. (2024). Preventing mosquito bites. Available online at: https://www.cdc.gov/mosquitoes/prevention/index.html (Accessed July 9, 2024).

[25] Wong PF Wong LP AbuBakar S. Diagnosis of severe dengue: challenges, needs and opportunities. J Infect Public Health. (2020) 13:193–8. doi: 10.1016/j.jiph.2019.07.012, PMID: 31405788

[26] Hung TM Shepard DS Bettis AA Nguyen HA McBride A Clapham HE. Productivity costs from a dengue episode in Asia: a systematic literature review. BMC Infect Dis. (2020) 20:393. doi: 10.1186/s12879-020-05109-0, PMID: 32493234 PMC7268537

[27] Luh DL Liu CC Luo YR Chen SC. Economic cost and burden of dengue during epidemics and non-epidemic years in Taiwan. J Infect Public Health. (2018) 11:215–23. doi: 10.1016/j.jiph.2017.07.021, PMID: 28757293

[28] Sitaresmi MN Seale H Heywood AE Padmawati RS Soenarto Y MacIntyre CR. Maternal knowledge and attitudes towards rotavirus diarrhea and vaccine acceptance in Yogyakarta, Indonesia: a qualitative study. Paediatr Indones. (2022) 62:333–40. doi: 10.14238/pi62.5.2022.333-40

[29] Romate J Rajkumar E Gopi A Abraham J Rages J Lakshmi R. What contributes to COVID-19 vaccine hesitancy? A systematic review of the psychological factors associated with COVID-19 vaccine hesitancy. Vaccine. (2022) 10:1777. doi: 10.3390/vaccines10111777, PMID: 36366286 PMC9698528

[30] Lavanya KM Kumar Andey UVV Mishra SK Vutharkar NR. Knowledge and practice about dengue fever among urban slum dwellers in one district of Andhra Pradesh, India: a study on current status. MRIMS J Health Sci. (2022) 10:93–8. doi: 10.4103/mjhs.mjhs_17_22

[31] Strategi Nasional (STRANAS) Penanggulangan Dengue. (2021–2025). Center for Tropical Medicine UGM. Available online at: https://centertropmed-ugm.org/project/stranas-dengue/ (Accessed June 6, 2024).

[32] Padde JR Lu Q Long Y Zhang D Hou M Chen L. The impact of environmental and host factors on *wolbachia* density and efficacy as a biological tool. Decod Infect Transm. (2023) 1:100006. doi: 10.1016/j.dcit.2023.100006, PMID: 40292123

[33] Opel DJ Salmon DA Marcuse EK. Building trust to achieve confidence in COVID-19 vaccines. JAMA Netw Open. (2020) 3:e2025672. doi: 10.1001/jamanetworkopen.2020.25672, PMID: 33079194

[34] Brandoctors. Badan POM Menyetujui Izin Edar Vaksin Dengue di Indonesia|Badan Pengawas Obat dan Makanan. Available online at: https://www.pom.go.id (Accessed June 11, 2024).

[35] Brandoctors. Penjelasan Publik|Badan Pengawas Obat dan Makanan. Available online at: https://www.pom.go.id (Accessed June 11, 2024).

[36] Takeda’s QDENGA®▼ (dengue tetravalent vaccine [live, attenuated]) approved in Indonesia for use regardless of prior dengue exposure. Available online at: https://www.takeda.com/newsroom/newsreleases/2022/takedas-qdenga-dengue-tetravalent-vaccine-live-attenuated-approved-in-indonesia-for-use-regardless-of-prior-dengue-exposure/ (Accessed April 29, 2024).

[37] Takeda announces WHO SAGE recommendation for dengue vaccine. Available online at: https://www.takeda.com/newsroom/newsreleases/2023/Takeda-Dengue-Vaccine-Recommended-by-World-Health-Organization-Advisory-Group-for-Introduction-in-High-Dengue-Burden-and-Transmission-Areas-in-Children-Ages-Six-to-16-Years/ (Accessed April 24, 2024).

[38] Sitaremi MN Soedjatmiko S Gunardi H Kaswandani N Handryastuti S Raihan R. Jadwal Imunisasi Anak Usia 0 – 18 Tahun Rekomendasi Ikatan Dokter Anak Indonesia Tahun 2023. Sari Pediatri. (2023) 25:64–74. doi: 10.14238/sp25.1.2023.64-74

[39] Vilar-Lluch S McClaughlin E Knight D Adolphs S Nichele E. The language of vaccination campaigns during COVID-19. Med Humanit. (2023) 49:487–96. doi: 10.1136/medhum-2022-012583, PMID: 37024299 PMC10511959

[40] Amelia VL Setiawan A Sukihananto S Amelia VL Setiawan A Sukihananto S. El juego de mesa como medio educativo para el conocimiento sobre la prevención del dengue en niños en edad escolar. Enferm Glob. (2019) 18:254–72. doi: 10.6018/eglobal.18.4.336611

[41] Minarti M Anwar C Irfannuddin I Irsan C. Community knowledge and attitudes about the transmission of dengue Haemorrhagic fever and its relationship to prevention behaviour in Palembang, South Sumatra, Indonesia. Open access Maced. J Med Sci. (2021) 9:1534–43. doi: 10.3889/oamjms.2021.7693, PMID: 32996903

[42] Kosasih CE Lukman M Solehati T Mediani HS. Effect of dengue hemorrhagic fever health education on knowledge and attitudes, in elementary school children in West Java, Indonesia. Linguist Cult Rev. (2021) 5:191–200. doi: 10.21744/lingcure.v5nS1.1349

[43] Udoudom U Igiri A George K Aruku K. Promoting health education through effective communication for development. Alsystech J Educ Technol. (2023) 2:68–88. doi: 10.58578/alsystech.v2i1.2399

[44] Evolving role of social Media in Health Promotion: Updated responsibilities for health education specialists. []. Available from: https://www.mdpi.com/1660-4601/17/4/1153 10.3390/ijerph17041153 PMC7068576 32059561

[45] Sultan MI Amir AS. The utilization of digital Media in Health Communication in Indonesia. AICCON. (2024) 1:408–18.

[46] Lubis TA Gunardi H Herqutanto SS Satari HI Alatas FS. Educational videos to address vaccine hesitancy in childhood immunization. Vaccine. (2022) 40:5965–70. doi: 10.1016/j.vaccine.2022.08.044, PMID: 36085255 PMC9446135

[47] Shafie AA Moreira ED Jr Vidal G Di Pasquale A Green A Tai R. Sustainable dengue prevention and management: integrating dengue vaccination strategies with population perspectives. Vaccine. (2024) 12:184. doi: 10.3390/vaccines12020184, PMID: 38400167 PMC10892244

[48] Global Strategy for Dengue Prevention and Control. (2012–2020). World Health Organization. 20. Available online at: https://iris.who.int/bitstream/handle/10665/75303/9789241504034_eng.pdf?sequence=1

[49] Nguyen-Tien T Probandari A Ahmad RA. Barriers to engaging communities in a dengue vector control program: an implementation research in an urban area in Hanoi city, Vietnam. Am J Trop Med Hyg. (2019) 100:964–73. doi: 10.4269/ajtmh.18-0411, PMID: 30652660 PMC6447129

[50] Sim S Ng LC Lindsay SW Wilson AL. A greener vision for vector control: the example of the Singapore dengue control programme. PLoS Negl Trop Dis. (2020) 14:e0008428. doi: 10.1371/journal.pntd.0008428, PMID: 32853197 PMC7451545

[51] Loftus R Sahm LJ Fleming A. A qualitative study of the views of healthcare professionals on providing vaccines information to patients. Int J Clin Pharm. (2021) 43:1683–92. doi: 10.1007/s11096-021-01299-y, PMID: 34155584 PMC8216584

[52] Hwang J. Health information sources and the influenza vaccination: the mediating roles of perceived vaccine efficacy and safety. J Health Commun. (2020) 25:727–35. doi: 10.1080/10810730.2020.1840675, PMID: 33186091

[53] Nikic P Stankovic B Santric V Vukovic I Babic U Radovanovic M. Role of healthcare professionals and sociodemographic characteristics in COVID-19 vaccination acceptance among Uro-oncology patients: a cross-sectional observational study. Vaccine. (2023) 11:911. doi: 10.3390/vaccines11050911, PMID: 37243015 PMC10222021

[54] Skafle I Nordahl-Hansen A Quintana DS Wynn R Gabarron E. Misinformation about COVID-19 vaccines on social media: rapid review. J Med Internet Res. (2022) 24:e37367. doi: 10.2196/37367, PMID: 35816685 PMC9359307

[55] Pavlovic D Sahoo P Larson HJ Karafillakis E. Factors influencing healthcare professionals’ confidence in vaccination in Europe: a literature review. Hum Vaccin Immunother. (2022) 18:2041360. doi: 10.1080/21645515.2022.2041360, PMID: 35290160 PMC9009961

