# Supplementary material for: Addressing knowledge, attitude and practice gaps for effective dengue management strategies in Indonesia
Source: Front Public Health. 2025 May 23;13:1540121. doi: 10.3389/fpubh.2025.1540121 (PMC12141280; doi:10.3389/fpubh.2025.1540121)
Supplement: Supplementary file 1 [file Table_1.docx]

# **SUPPLEMENTARY MATERIALS**

**Supplementary Table 1:** Definition and derivation of Knowledge, Attitude, and Practice sub-categories of KAP framework

|  | **Sub-category** | **Definition** | **Calculation** |
| --- | --- | --- | --- |
| **Knowledge** | Dengue infection and symptoms | Knowledge level on dengue transmission methods, serotypes of dengue, and diagnosis methods | Percentage of correct answers given by the respondent on questions regarding dengue disease and vector control |
|  | Dengue vector control methods and vaccines | Knowledge level on dengue vector control prevention (methods and recommended frequency) and vaccines (vaccine safety and effectiveness; government, physician, or community leader endorsement; and dengue vaccine availability) |  |
| **Attitude** | Dengue infection and symptoms | Attitudes regarding dengue disease (likelihood of contracting dengue, threat, Attitude toward government response) and severity of symptoms | Mean value of all Attitude-related questions on the Likert scale (0-10) |
|  | Dengue prevention methods | Attitudes regarding dengue prevention (perceived effectiveness and safety of dengue vector control prevention measures, adherence to prevention measures at a societal level) |  |
|  | Vaccines | Attitudes regarding vaccination (factors that positively and negatively impacted decisions about vaccination, Attitudes toward the dengue vaccine at a personal and a societal level) |  |
| **Practice** | Dengue prevention methods | Practice of dengue prevention focused on the number of and level of confidence in dengue prevention/ vector control measures performed by the individual | A function of the number of prevention methods listed by the respondent and the level of confidence ascribed to the prevention methods conducted |

**Supplementary Figure 1**: Attitude regarding general vaccines in Indonesia

**
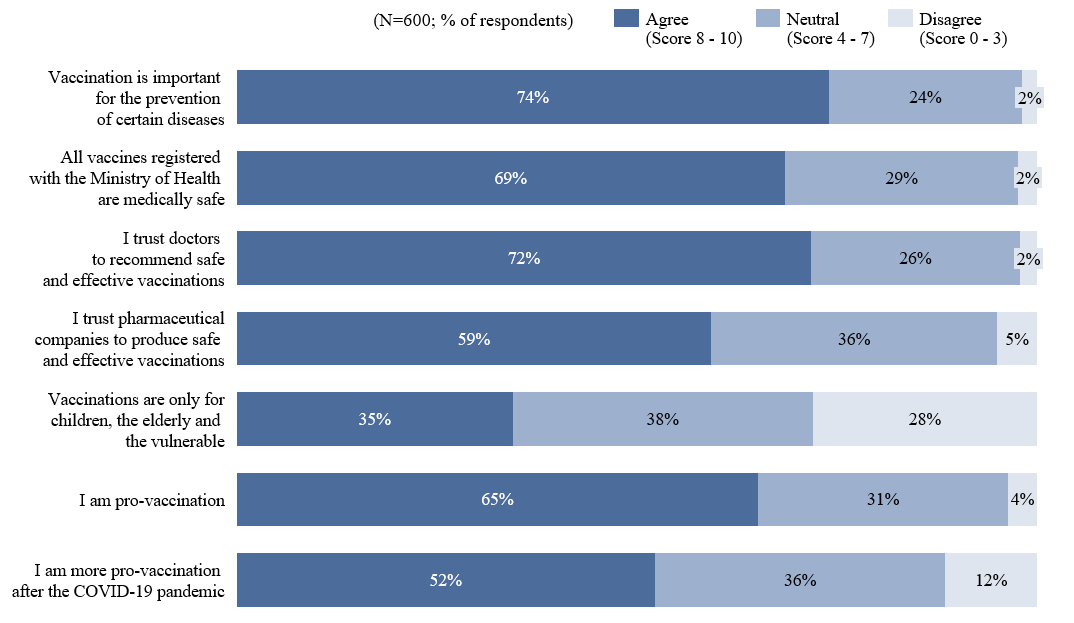
**

**Supplementary Figure 2:** Preferred sources for seeking health information

***
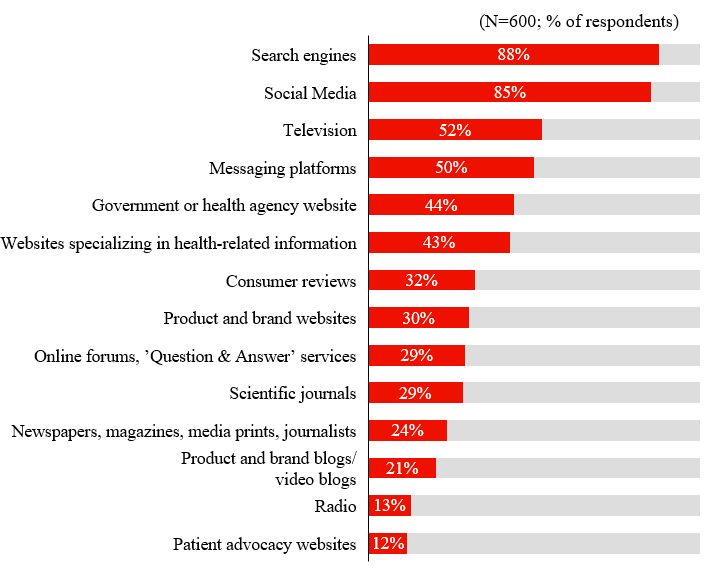
***

**Supplementary Figure 3:** Trusted stakeholders for health-related information

**
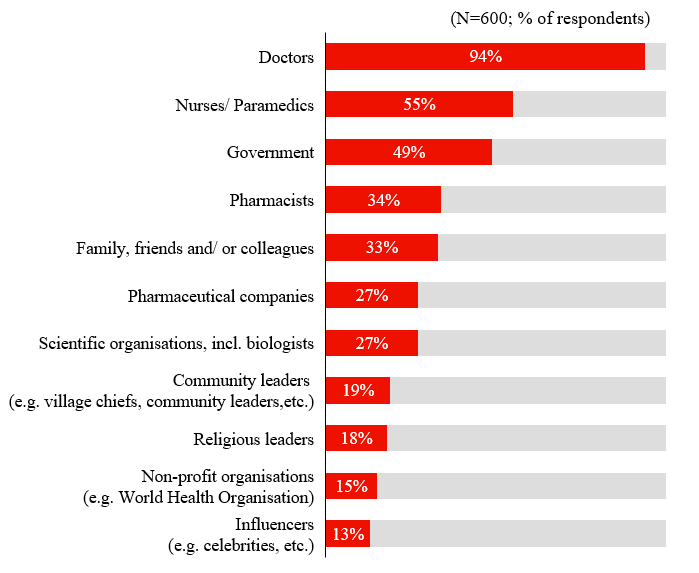
**

**Supplementary Figure 4**: Preference for approaches toward nationwide dengue management

**
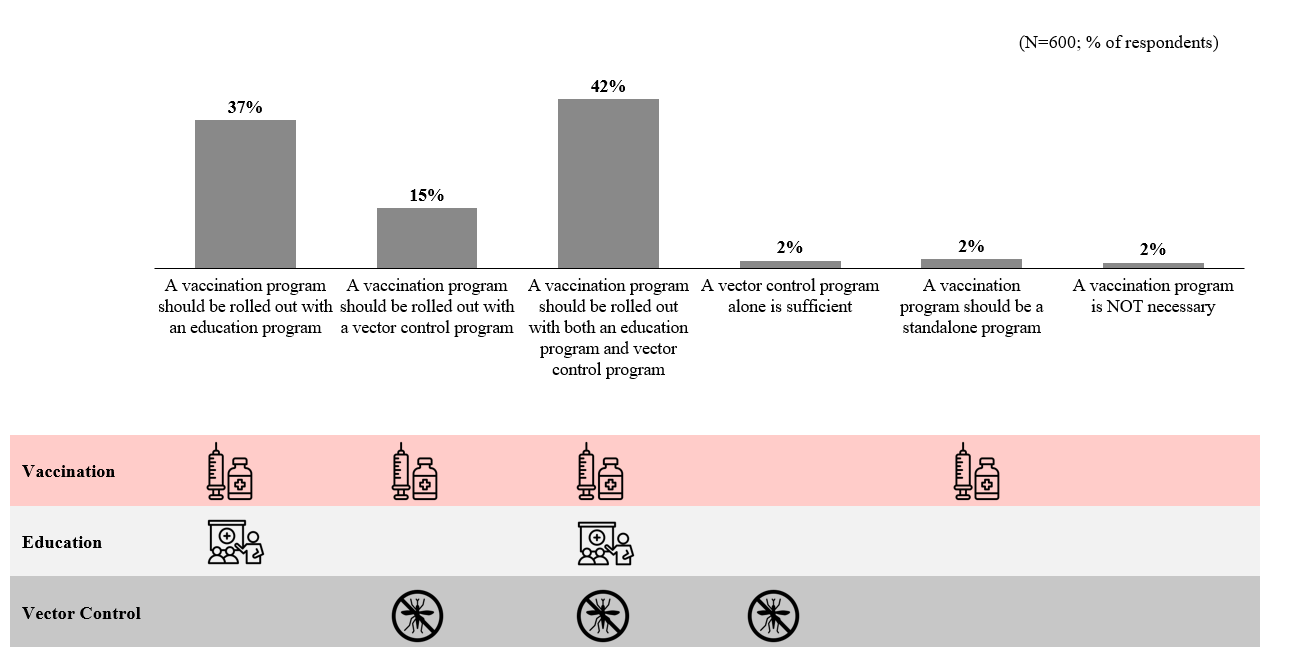
**

**Supplementary File 1** GEMKAP Dengue Study Screener and Main Questionnaire

# **GEMKAP DENGUE STudy Screener and Main SURVEY (Global)**

# **1.SCREENER**

**GENDER**

1. **What is your gender?**

- Male
- Female

**AGE**

1. **How old were you at your last birthday? Please enter your age in years.**

- Younger than 17 years old
- 17-30 years old
- 31-40 years old
- 41-50 years old
- 51-60 years old
- Older than 60 years old

**HOUSEHOLD SIZE**

1. **How many people are living in your household including yourself?**

*By household we mean the number of people (including yourself) who live at the same address, who share cooking facilities and share a living room or sitting room or dining area.*

- I live alone
- 1-2 members
- 3-4 members
- 5-6 members
- >6 members

1. **Are you a parent? If yes, how many children (below legal age) do you have?**

- No children
- 1-2 children
- 3-4 children
- >4 children

1. **Are you currently pregnant?**

If FEMALE is selected in S2;

- Yes
- No
- I don’t know

**ETHNICITY**

1. **Which of these best describes your ethnicity?**

- Amerindian/Native American
- African
- Betawi
- Bumiputera
- Chinese
- Indian
- Javanese
- Madurese
- Malay
- Mestizo and White/European
- Others

**RELIGION**

1. **Which of these best describes your religion?**

- Buddhism or Taoism
- Christianity
- Islam
- Hinduism
- Others
- No religion

**REGION**

1. **In which region do you live in?**

- Argentina – Buenos Aires
- Argentina – Central
- Argentina – Cuyo
- Argentina – Patagonia
- Argentina – North-west
- Argentina – North-east
- Brazil – North
- Brazil – North-east
- Brazil – Central west
- Brazil – South-east
- Brazil – South
- Colombia – Amazon Region
- Colombia – Andean Region
- Colombia – Caribbean Region
- Colombia – Orinoquía Region
- Colombia – Pacific Region
- Mexico – North-west
- Mexico – North-east
- Mexico – Central north
- Mexico – Central south
- Mexico – West
- Mexico – East
- Mexico – South-west
- Mexico – South-east
- Indonesia – Sumatra
- Indonesia – Java
- Indonesia – Kalimantan
- Indonesia – Sulawesi
- Indonesia – Lesser Sunda Islands
- Indonesia – Maluku & Papua
- Malaysia – Northern
- Malaysia – East Coast
- Malaysia – Central
- Malaysia – Southern
- Malaysia – Eastern (Sarawak)
- Malaysia – Eastern (Sabah)
- Singapore – Central
- Singapore – East
- Singapore – North
- Singapore – North-east
- Singapore – West

1. **In which cities/ districts do you live in?**

- Brazil – Belo Horizonte
- Brazil – Campo Grande
- Brazil – Petrolina
- Brazil – Niteroi
- Brazil – Rio de Janeiro
- Brazil – Others
- Colombia – Bello
- Colombia – Cali
- Colombia – Itagui
- Colombia – Medellin
- Colombia – Others
- Mexico – La Paz
- Mexico – Others
- Indonesia – Yogyakarta
- Indonesia – Others
- Malaysia – Kelantan
- Malaysia – Kuala Lumpur
- Malaysia – Putrajaya
- Malaysia – Penang
- Malaysia – Selangor
- Malaysia – Others
- Singapore – Bukit Batok
- Singapore – Choa Chu Kang
- Singapore – Tampines
- Singapore – Yishun
- Singapore – Others

**EDUCATION**

1. **What is your highest educational qualification?**

- No formal education
- Primary education only, e.g. Primary school, elementary school
- Secondary education, e.g. Secondary school, middle school
- Tertiary education, e.g. College, bachelors, university-level, trade school
- Post tertiary education, e.g. Masters, PhD

**DENGUE**

1. **Have you contracted Dengue/Breakbone disease previously?**

- Yes
- No

1. **Have you taken part in a Dengue/Breakbone disease survey in the past 3 months?**

- Yes
- No
- I’m not sure

**VACCINATION**

1. **Are you a decision maker for your health?**

- Yes, I am personally responsible for my health
- Yes, but I share the responsibility with someone else (e.g. Family, caregiver)
- No, someone else (e.g. Family, caregiver) is making the decision for me.

1. **Do you think vaccines are useful?**

- YES, I have full confidence in most vaccines
- YES, I have some confidence in most vaccines
- YES, but I am hesitant about vaccines
- I have no opinions about vaccines
- NO, but I willing to consider vaccines
- NO, I don’t believe vaccines are useful
- NO, I am strongly against vaccines, regardless of government approval

1. **Out of the optional vaccines below which vaccinations have you taken? Select all that apply.**

- Dengue
- COVID-19
- Cholera
- Hepatitis A
- Hepatitis B
- HPV (Human Papillomavirus)
- Influenza
- Japanese Encephalitis
- Measles, Mumps and Rubella
- Meningitis (Meningococcal Vaccine)
- Rabies
- Tdap (Tetanus, Diphtheria and Pertussis)
- Tickborne Encephalitis
- Typhoid
- Varicella (Chicken Pox, Shingles Vaccine)
- Yellow Fever
- None of the above

**INCOME**

1. **And finally, which range best describes your MONTHLY HOUSEHOLD INCOME**

**Please consider ALL sources of revenue (including personal income, money sent from family overseas, revenue from investments, etc.)**

- Argentina (ARS) - Low (<30,000)
- Argentina (ARS) - Mid (30,000 – 79,999)
- Argentina (ARS) - High (≥80,000)
- Brazil (BRL) - Low (<2,000)
- Brazil (BRL) - Mid (2,000 – 6,999)
- Brazil (BRL) - High (≥7,000)
- Colombia (COP) - Low (<900,000,000)
- Colombia (COP) - Mid (900,000 – 2,699,999)
- Colombia (COP) - High (≥2,700,000)
- Mexico (MXP) - Low (<9,000)
- Mexico (MXP) - Mid (9,000 – 39,999)
- Mexico (MXP) - High (≥40,000)
- Indonesia (IDR) – Low (<1,500,000)
- Indonesia (IDR) – Mid (1,500,000 – 4,999,999)
- Indonesia (IDR) – High (≥5,000,000)
- Malaysia (MYR) – Low (<5,000)
- Malaysia (MYR) – Mid (5,000 – 10,999)
- Malaysia (MYR) – Mid (11,000 – 10,999)
- Malaysia (MYR) – Mid (≥11,000)
- Singapore (SGD) – Low (<3,000)
- Singapore (SGD) – Mid (3,000 – 12,499)
- Singapore (SGD) – High (≥12,500)

# **2.MAIN STUDY SURVEY**

**DENGUE KNOWLEDGE**

**Q1. Please select true or false for the following statements.**

|  | True | False |
| --- | --- | --- |
| 1. Dengue is transmitted to a person via Aedes mosquitoes |  |  |
| 1. Aedes mosquitoes are more likely to bite in the evenings or at night |  |  |
| 1. Mosquitoes reproduce OR multiply in stagnant water |  |  |
| 1. Mosquitoes reproduce OR multiply in clear water |  |  |
| 1. Mosquitoes are more likely to bite when the weather is hot |  |  |
| 1. Mosquitoes are more likely to bite in humid weather. (Humidity refers to the amount of water vapor in the air. The higher the humidity, the higher the moisture in the air and the wetter it feels outside.) |  |  |

**Q2. Please select true or false for the following statements.**

|  | True | False |
| --- | --- | --- |
| 1. I live in an area where dengue is very common |  |  |
| 1. There are 4 different virus types of dengue |  |  |
| 1. You can only catch dengue once in your lifetime |  |  |
| 1. You can only catch dengue once in your lifetime |  |  |
| 1. I may be infected by 1 or more virus types of dengue at different points of time |  |  |
| 1. I cannot be infected by 2 or more virus types of dengue at the same time |  |  |
| 1. People can die from dengue and its related complications (e.g. fever, cough etc.) |  |  |
| 1. It is always possible to tell when someone has dengue by looking at them |  |  |
|  |  |  |

**Q3. Have you or anyone else you know contracted dengue two times or more?**

- Yes, I have contracted dengue at least two times or more
- Yes, I know someone who has contracted dengue at least two times or more
- No, I do not know anyone who has contracted dengue at least two times or more

**Q4. How severe is dengue as a disease? Please rate each statement on a scale from 0 to 10, with 0 being 'not severe at all' and 10 being 'very severe'**

|  | (0) | (1) | (2) | (3) | (4) | (5) | (6) | (7) | (8) | (9) | (10) |  |
| --- | --- | --- | --- | --- | --- | --- | --- | --- | --- | --- | --- | --- |
| Not severe at all |  |  |  |  |  |  |  |  |  |  |  | Very severe |

**Q5. Thinking about yourself and your family, community and neighborhood, how likely is it that the following people will contract dengue?**

**Please rate each statement on a scale from 0 to 10, with 0 being 'very unlikely' and 10 being 'very likely'.**

|  |  | (0) | (1) | (2) | (3) | (4) | (5) | (6) | (7) | (8) | (9) | (10) |  |
| --- | --- | --- | --- | --- | --- | --- | --- | --- | --- | --- | --- | --- | --- |
| 1. Yourself | Very unlikely |  |  |  |  |  |  |  |  |  |  |  | Very likely |
| 1. Family | Very unlikely |  |  |  |  |  |  |  |  |  |  |  | Very likely |
| 1. Older residents who live in the neighborhood, aged 65 years old | Very unlikely |  |  |  |  |  |  |  |  |  |  |  | Very likely |
| 1. People with chronic or long-term health conditions | Very unlikely |  |  |  |  |  |  |  |  |  |  |  | Very likely |
| 1. Infants, children and adolescents of up to 18 years old | Very unlikely |  |  |  |  |  |  |  |  |  |  |  | Very likely |
| 1. Adult residents, aged 18 - 65 years | Very unlikely |  |  |  |  |  |  |  |  |  |  |  | Very likely |
| 1. Anyone | Very unlikely |  |  |  |  |  |  |  |  |  |  |  | Very likely |

**Q6. How is dengue diagnosed? Please select all that apply.**

- Self-testing blood kit performed at home
- Self-testing nasal swab kit performed at home
- Blood test at the clinic or hospital
- Medical scans
- Signs/symptoms of the disease e.g. sore throat, cough, fever etc.
- Previous medical and travel history
- I do not know

**Q7. Which of the following can often be signs of dengue? Please select all that apply**

- Fever
- Headache
- Runny nose
- Flushing (face and/or body becomes red)
- Body aches/joint and muscle pain
- Swollen glands and lymph nodes
- Body rashes
- Body chills
- Hair loss (alopecia)
- Feeling very tired and sleepy
- Difficulty breathing
- Nausea and vomiting
- Loss of appetite
- Abdominal/ belly / stomach pain
- Pain behind the eyes
- Diarrhoea
- Bleeding from nose or gums
- All of the above

**Q8. If someone were to contract dengue, what might the consequences be? Please select all that apply.**

- Absenteeism from school/work
- Potential clinic visits
- Potential hospitalization
- Additional unexpected treatment costs from stay at hospital e.g. blood tests
- Additional costs due to caregiver requirements (incl. absence from work)
- Worsening of chronic and long-term health conditions
- Increased risk of contracting dengue again
- Increasing severity of dengue should it be contracted again
- Poorer quality of life
- All of the above

**Q9. How much do you agree with each of the following statements?**

**Please rate each statement on a scale from 0 to 10, with 0 being strongly disagree’ and 10 being ‘strongly agree'.**

|  |  | (0) | (1) | (2) | (3) | (4) | (5) | (6) | (7) | (8) | (9) | (10) |  |
| --- | --- | --- | --- | --- | --- | --- | --- | --- | --- | --- | --- | --- | --- |
| 1. There is nothing we can do to treat dengue | Strongly disagree |  |  |  |  |  |  |  |  |  |  |  | Strongly agree |
| 1. There is nothing we can do to prevent dengue | Strongly disagree |  |  |  |  |  |  |  |  |  |  |  | Strongly agree |
| 1. The threat of dengue is or has been exaggerated by the media | Strongly disagree |  |  |  |  |  |  |  |  |  |  |  | Strongly agree |
| 1. The threat of dengue is or has been exaggerated by the government | Strongly disagree |  |  |  |  |  |  |  |  |  |  |  | Strongly agree |
| 1. The government is responding appropriately to combat dengue | Strongly disagree |  |  |  |  |  |  |  |  |  |  |  | Strongly agree |
| 1. The government is well prepared to combat dengue | Strongly disagree |  |  |  |  |  |  |  |  |  |  |  | Strongly agree |
| 1. We will all be completely powerless | Strongly disagree |  |  |  |  |  |  |  |  |  |  |  | Strongly agree |
| 1. We just have to accept it | Strongly disagree |  |  |  |  |  |  |  |  |  |  |  | Strongly agree |

**DENGUE PREVENTION**

**Q10. Based on your current knowledge about dengue, do you think the following statements are true or false?**

- There are specific medicines that can cure dengue
- There is no vaccine yet that can prevent dengue
- There is a vaccine that can prevent dengue but it is not yet registered in my country

**Q11. Which of the activities below are you currently practicing to prevent the transmission of dengue?**

**Please select all that apply.**

- Spray insect repellent and/ or apply mosquito repellent patches
- Participate in community mosquito fogging
- Wear long-sleeved shirts and/ or long pants
- Use wire mesh mosquito screens and/ or mosquito nets
- Throw out any open bodies of water in plant containers, flower pots, tyres etc.
- Perform periodic maintenance of water tanks
- Tightly cover all water containers
- Keep drain free of blockage
- Place all garbage that can accumulate water into a closed bin
- Add larvicide in water containers to kill mosquito larvae
- None of the above

**Q12**. **How often do you complete these dengue prevention activities? Please select the answer closest to what you do.**

- Always/ nearly all the time
- Every few hours/ at least twice a day
- Once a day
- Once every other day
- Once a week
- Once every two weeks
- Once a month
- Less than once a month
- As and when needed
- None of the above

**Q13**. **How confident are you in completing these dengue prevention activities successfully? Please rate each statement on a scale from 0 to 10, with 0 being 'not confident at all' and 10 being 'very confident'.**

|  |  | (0) | (1) | (2) | (3) | (4) | (5) | (6) | (7) | (8) | (9) | (10) |  |
| --- | --- | --- | --- | --- | --- | --- | --- | --- | --- | --- | --- | --- | --- |
| 1. Use wire mesh screens, mosquito nets and/or mosquito coils | Not confident at all |  |  |  |  |  |  |  |  |  |  |  | Very confident |
| 1. Drain water from pots and cover all water containers | Not confident at all |  |  |  |  |  |  |  |  |  |  |  | Very confident |
| 1. Community mosquito fogging | Not confident at all |  |  |  |  |  |  |  |  |  |  |  | Very confident |
| 1. Spraying mosquito repellent and using larvicide to kill mosquito larvae | Not confident at all |  |  |  |  |  |  |  |  |  |  |  | Very confident |
| 1. Wolbachia program (WIAM) | Not confident at all |  |  |  |  |  |  |  |  |  |  |  | Very confident |
| 1. Dengue vaccination | Not confident at all |  |  |  |  |  |  |  |  |  |  |  | Very confident |

**Q14**. **How effective do you think each of the dengue prevention methods is for your personal health? Please rate each statement on a scale from 0 to 10, with 0 being 'not confident at all' and 10 being 'very confident'.**

|  |  | (0) | (1) | (2) | (3) | (4) | (5) | (6) | (7) | (8) | (9) | (10) |  |
| --- | --- | --- | --- | --- | --- | --- | --- | --- | --- | --- | --- | --- | --- |
| 1. Use wire mesh screens, mosquito nets and/or mosquito coils | Not safe at all |  |  |  |  |  |  |  |  |  |  |  | Very safe |
| 1. Drain water from pots and cover all water containers | Not safe at all |  |  |  |  |  |  |  |  |  |  |  | Very safe |
| 1. Community mosquito fogging | Not safe at all |  |  |  |  |  |  |  |  |  |  |  | Very safe |
| 1. Spraying mosquito repellent and using larvicide to kill mosquito larvae | Not safe at all |  |  |  |  |  |  |  |  |  |  |  | Very safe |
| 1. Wolbachia program (WIAM) | Not safe at all |  |  |  |  |  |  |  |  |  |  |  | Very safe |
| 1. Dengue vaccination | Not safe at all |  |  |  |  |  |  |  |  |  |  |  | Very safe |

**Q15**. **How safe do you think each of each of the following dengue prevention methods is for your personal health?**

**Please rate each statement on a scale from 0 to 10, with 0 being ‘not safe at all’ and 10 being ‘very safe’.**

|  |  | (0) | (1) | (2) | (3) | (4) | (5) | (6) | (7) | (8) | (9) | (10) |  |
| --- | --- | --- | --- | --- | --- | --- | --- | --- | --- | --- | --- | --- | --- |
| 1. Use wire mesh screens, mosquito nets and/or mosquito coils | Not safe at all |  |  |  |  |  |  |  |  |  |  |  | Very safe |
| 1. Drain water from pots and cover all water containers | Not safe at all |  |  |  |  |  |  |  |  |  |  |  | Very safe |
| 1. Community mosquito fogging | Not safe at all |  |  |  |  |  |  |  |  |  |  |  | Very safe |
| 1. Spraying mosquito repellent and using larvicide to kill mosquito larvae | Not safe at all |  |  |  |  |  |  |  |  |  |  |  | Very safe |
| 1. Wolbachia program (WIAM) | Not safe at all |  |  |  |  |  |  |  |  |  |  |  | Very safe |
| 1. Dengue vaccination | Not safe at all |  |  |  |  |  |  |  |  |  |  |  | Very safe |

**Q16**. **How likely are you, your community/ neighborhood/ local council leader(s) or your government leader(s) (e.g. governors, mayors, councilors, etc.) to do the following dengue prevention activities in the next 6 months?**

**Please rate each statement on a scale from 0 to 10, with 0 being 'very unlikely' and 10 being 'very likely'.**

|  |  | (0) | (1) | (2) | (3) | (4) | (5) | (6) | (7) | (8) | (9) | (10) |  |
| --- | --- | --- | --- | --- | --- | --- | --- | --- | --- | --- | --- | --- | --- |
| 1. Use wire mesh screens, mosquito nets and/or mosquito coils | Very unlikely |  |  |  |  |  |  |  |  |  |  |  | Very likely |
| 1. Drain water from pots and cover all water containers | Very unlikely |  |  |  |  |  |  |  |  |  |  |  | Very likely |
| 1. Community mosquito fogging | Very unlikely |  |  |  |  |  |  |  |  |  |  |  | Very likely |
| 1. Spraying mosquito repellent and using larvicide to kill mosquito larvae | Very unlikely |  |  |  |  |  |  |  |  |  |  |  | Very likely |
| 1. Wolbachia program (WIAM) | Very unlikely |  |  |  |  |  |  |  |  |  |  |  | Very likely |
| 1. Dengue vaccination | Very unlikely |  |  |  |  |  |  |  |  |  |  |  | Very likely |

**DENGUE PREVENTION**

**Q17. How much do you agree with the statements below on vaccines as a preventative activity in general?**

**Please rate each statement on a scale from 0 to 10, with 0 being 'strongly disagree' and 10 being 'strongly agree'.**

|  |  | (0) | (1) | (2) | (3) | (4) | (5) | (6) | (7) | (8) | (9) | (10) |  |
| --- | --- | --- | --- | --- | --- | --- | --- | --- | --- | --- | --- | --- | --- |
| 1. Vaccination is important for the prevention of certain diseases | Strongly disagree |  |  |  |  |  |  |  |  |  |  |  | Strongly agree |
| 1. All vaccines registered with the Ministry of Health are medically safe | Strongly disagree |  |  |  |  |  |  |  |  |  |  |  | Strongly agree |
| 1. I trust doctors to recommend safe and effective vaccinations | Strongly disagree |  |  |  |  |  |  |  |  |  |  |  | Strongly agree |
| 1. I trust pharmaceutical companies to produce safe and effective vaccinations | Strongly disagree |  |  |  |  |  |  |  |  |  |  |  | Strongly agree |
| 1. Vaccinations are only for children, the elderly and the vulnerable | Strongly disagree |  |  |  |  |  |  |  |  |  |  |  | Strongly agree |
| 1. I am pro-vaccination | Strongly disagree |  |  |  |  |  |  |  |  |  |  |  | Strongly agree |
| 1. I am more pro-vaccination after the COVID-19 pandemic | Strongly disagree |  |  |  |  |  |  |  |  |  |  |  | Strongly agree |

**Q18. Based on your past vaccination experience (in general), how much do you agree with the statements below?**

**Please rate each statement on a scale from 0 to 10, with 0 being 'strongly disagree' and 10 being 'strongly agree'.**

|  |  | (0) | (1) | (2) | (3) | (4) | (5) | (6) | (7) | (8) | (9) | (10) |  |
| --- | --- | --- | --- | --- | --- | --- | --- | --- | --- | --- | --- | --- | --- |
| 1. My doctor recommends my family and I vaccines for several health conditions as appropriate. | Strongly disagree |  |  |  |  |  |  |  |  |  |  |  | Strongly agree |
| 1. I make sure that my children's vaccination schedule is followed | Strongly disagree |  |  |  |  |  |  |  |  |  |  |  | Strongly agree |
| 1. I proactively ask my family's or children's doctor about vaccinations | Strongly disagree |  |  |  |  |  |  |  |  |  |  |  | Strongly agree |
| 1. I receive reminders from doctors/ the government about my upcoming vaccinations | Strongly disagree |  |  |  |  |  |  |  |  |  |  |  | Strongly agree |
| 1. The government has broadcasted education campaigns for people to get vaccinated | Strongly disagree |  |  |  |  |  |  |  |  |  |  |  | Strongly agree |
| 1. The government has made it easy for people to get vaccinated by offering it at convenient locations | Strongly disagree |  |  |  |  |  |  |  |  |  |  |  | Strongly agree |
| 1. My community/ government leader(s) (e.g. governors, mayors, councilors etc.) promotes the importance of vaccines | Strongly disagree |  |  |  |  |  |  |  |  |  |  |  | Strongly agree |
| 1. My favorite influencer(s) (e.g. local and international celebrities, etc.) promotes the importance of vaccines | Strongly disagree |  |  |  |  |  |  |  |  |  |  |  | Strongly agree |
| 1. It is easy to schedule a vaccination appointment | Strongly disagree |  |  |  |  |  |  |  |  |  |  |  | Strongly agree |

**Q19. Based on your past vaccination experience, please select all the statements that apply to your decision to take the influenza vaccine.**

- The vaccination was free, discounted or the cost was claimed back from the government or insurance company
- I received an incentive (cash, points, or a present) to take the vaccine
- There was sufficient scientific evidence on the vaccine's safety and effectiveness
- I was aware of the consequences if I did not vaccinate
- I was not afraid of the vaccine's side effects
- The vaccine's benefits outweigh the side effects
- I felt that I was at high risk of contracting influenza
- It contributed to herd immunity (where a large part of the population is immune to influenza, reducing the spread of the disease in the general population)
- It was recommended by the government
- It was recommended by my doctor
- It is easy to get a vaccination appointment
- I heard about a friend/ family who had developed severe symptoms from influenza
- I wanted to protect my friends and family from contracting influenza
- My friends and family encouraged me to receive the vaccine
- I was given the vaccine during a routine visit to see a doctor
- **Others: ___**

**Q20. Please select all the statements that apply to you not receiving the influenza vaccine.**

- The vaccination was not free/ discounted/ claimed from the government or insurance company
- The vaccination was not affordable
- There was no incentive (cash, points, or a gift) to take the vaccine
- There was not sufficient scientific evidence on the vaccine's safety and how well it protects against influenza
- There are no consequences if I did not vaccinate
- I think that the vaccine is not safe
- I was afraid of the side effects that I might experience from the vaccine
- I experienced a side effect(s) from the vaccine and am reluctant to receive further vaccination
- The vaccines' side effects outweighed its benefits
- I do not think that I was at risk of contracting influenza
- It does not contribute to herd immunity (where a large part of the population is immune to influenza, reducing the spread of the disease in the general population)
- It was not recommended by the government
- It was not recommended by my doctor
- It is not easy to get a vaccination appointment
- I heard about a friend/ family who had developed severe side effects from the influenza vaccine
- Even if I was sick with influenza, I do not think I would infect others with influenza
- My friends and family discouraged me to take the vaccine
- I have been previously advised against receiving a vaccination
- I am afraid of needles
- **Others: ___**

**Q21. Assuming there is a vaccine developed for dengue prevention that is approved by global and local health authorities, how much do you agree with the statements below? Please rate each statement on a scale from 0 to 10, with 0 being 'strongly disagree' and 10 being 'strongly agree'.**

|  |  | (0) | (1) | (2) | (3) | (4) | (5) | (6) | (7) | (8) | (9) | (10) |  |
| --- | --- | --- | --- | --- | --- | --- | --- | --- | --- | --- | --- | --- | --- |
| 1. I am concerned about the level of protection the vaccine will give me | Strongly disagree |  |  |  |  |  |  |  |  |  |  |  | Strongly agree |
| 1. I am concerned that I may need repeated booster vaccines to maintain immunity | Strongly disagree |  |  |  |  |  |  |  |  |  |  |  | Strongly agree |
| 1. I am concerned about the vaccine safety and adverse effects | Strongly disagree |  |  |  |  |  |  |  |  |  |  |  | Strongly agree |
| 1. I think vaccines are harmful | Strongly disagree |  |  |  |  |  |  |  |  |  |  |  | Strongly agree |
| 1. I am concerned that the vaccine will transmit the dengue virus to me | Strongly disagree |  |  |  |  |  |  |  |  |  |  |  | Strongly agree |
| 1. I do not want to be the first to try a new dengue vaccine | Strongly disagree |  |  |  |  |  |  |  |  |  |  |  | Strongly agree |
| 1. I will wait to be reassured that there are no safety risks | Strongly disagree |  |  |  |  |  |  |  |  |  |  |  | Strongly agree |
| 1. I am concerned that the vaccine may contain heavy metals or dangerous substances | Strongly disagree |  |  |  |  |  |  |  |  |  |  |  | Strongly agree |
| 1. I am concerned that the vaccine may cause autism | Strongly disagree |  |  |  |  |  |  |  |  |  |  |  | Strongly agree |
| 1. My immune system is weak and I believe that taking the vaccine will cause me more harm | Strongly disagree |  |  |  |  |  |  |  |  |  |  |  | Strongly agree |
| 1. I will consider it only for my children or parents | Strongly disagree |  |  |  |  |  |  |  |  |  |  |  | Strongly agree |
| 1. I am afraid of needles | Strongly disagree |  |  |  |  |  |  |  |  |  |  |  | Strongly agree |
| 1. I don't believe in vaccines | Strongly disagree |  |  |  |  |  |  |  |  |  |  |  | Strongly agree |

**Q22. How much do you agree with the statements below regarding a hypothetical dengue vaccine?
Please rate each statement on a scale from 0 to 10, with 0 being 'strongly disagree' and 10 being 'strongly agree'.**

|  |  | (0) | (1) | (2) | (3) | (4) | (5) | (6) | (7) | (8) | (9) | (10) |  |
| --- | --- | --- | --- | --- | --- | --- | --- | --- | --- | --- | --- | --- | --- |
| 1. I believe that the vaccine should be made accessible to the public including myself | Strongly disagree |  |  |  |  |  |  |  |  |  |  |  | Strongly agree |
| 1. I trust the healthcare system and professionals in my country to deliver the vaccine and manage its side effects | Strongly disagree |  |  |  |  |  |  |  |  |  |  |  | Strongly agree |
| 1. If I have to pay for the vaccination, I will not do it | Strongly disagree |  |  |  |  |  |  |  |  |  |  |  | Strongly agree |
| 1. I will be more willing to get vaccinated if there are incentives (cash, points, or a gift) | Strongly disagree |  |  |  |  |  |  |  |  |  |  |  | Strongly agree |
| 1. It depends on how severe the dengue epidemic is/ how common dengue is when I am offered the vaccine | Strongly disagree |  |  |  |  |  |  |  |  |  |  |  | Strongly agree |
| 1. If the risk of contracting dengue is low, I may not get the dengue vaccine | Strongly disagree |  |  |  |  |  |  |  |  |  |  |  | Strongly agree |
| 1. I think dengue vaccination is more important than other optional vaccines (e.g. influenza) | Strongly disagree |  |  |  |  |  |  |  |  |  |  |  | Strongly agree |
| 1. I am not convinced that it will be effective, look at the influenza/COVID-19 vaccine | Strongly disagree |  |  |  |  |  |  |  |  |  |  |  | Strongly agree |

**Q23. Assuming there is a vaccine developed for dengue prevention that is approved by global and local health authorities, how willing are you to consider getting vaccinated or recommend vaccination against dengue?**

**Please rate each statement on a scale from 0 to 10, with 0 being 'not willing at all' and 10 being 'very willing'.**

|  |  | (0) | (1) | (2) | (3) | (4) | (5) | (6) | (7) | (8) | (9) | (10) |  |
| --- | --- | --- | --- | --- | --- | --- | --- | --- | --- | --- | --- | --- | --- |
| 1. Myself | Not willing at all |  |  |  |  |  |  |  |  |  |  |  | Very willing |
| 1. My spouse (if applicable) | Not willing at all |  |  |  |  |  |  |  |  |  |  |  | Very willing |
| 1. My children (if applicable) | Not willing at all |  |  |  |  |  |  |  |  |  |  |  | Very willing |
| 1. My parents (if applicable) | Not willing at all |  |  |  |  |  |  |  |  |  |  |  | Very willing |
| 1. My friends | Not willing at all |  |  |  |  |  |  |  |  |  |  |  | Very willing |

**Q24. Are you willing to consider a dengue vaccine if it is recommended by your physician?
Please rate this statement on a scale from 0 to 10, with 0 being 'strongly disagree' and 10 being 'strongly agree'.**

|  | (0) | (1) | (2) | (3) | (4) | (5) | (6) | (7) | (8) | (9) | (10) |  |
| --- | --- | --- | --- | --- | --- | --- | --- | --- | --- | --- | --- | --- |
| Strongly disagree |  |  |  |  |  |  |  |  |  |  |  | Strongly agree |

**DENGUE VACCINATION + VECTOR CONTROL**

**Q25. Do you think that a dengue vaccination program should be rolled out with other programs, or as a standalone program?** *Other programs may include dengue vaccine education, vector control (any method to limit or eliminate mosquitoes, which transmit dengue), vector control education etc.*

- I believe that a vaccination program should be rolled out with an education program
- I believe that a vaccination program should be rolled out with a vector control program (any method to limit or eradicate mosquitoes which transmit dengue)
- I believe that a vaccination program should be rolled out with both an education program and vector control program
- I believe that a vector control program alone is sufficient
- I believe that a vaccination program should be a standalone program
- I don't believe a vaccination program is necessary

**Q26. Please share any other reason why you would be willing to get a dengue vaccine.**

|  |
| --- |

**Q27. Please share any other reason why you would not be willing to get a dengue vaccine.**

|  |
| --- |

**Q28. Please select which dengue prevention activities the government should allocate more resources to (e.g., funding, subsidizing, distribution, manpower) in the future.**

- Use wire mesh screens, mosquito nets or mosquito coils
- Drain water from pots and cover all water containers
- Community mosquito fogging
- Spraying mosquito repellent and using larvicide to kill mosquito larvae
- Wolbachia program (WIAM): Natural bacteria called Wolbachia reduce the abilities of Aedes mosquitoes to transmit dengue. Mosquitoes bred with the Wolbachia bacteria are released into nature to spread the bacteria among the wider mosquito population, thus naturally reducing the transmission of dengue.
- Dengue vaccination
- Others: ___
- None of the above

**EDUCATION**

**Q29. Do you agree with the statements below on the impact of religion on your daily decision-making process?**

**Please rate each statement on a scale from 0 to 10, with 0 being 'strongly disagree' and 10 being 'strongly agree'.**

*Religion and spirituality may impact decisions related to but not including diet, medicines with animal origins (e.g. halal vaccines, sterile insect treatments, blood transfusions etc.) and preferred gender of doctors or other healthcare professionals*

|  |  | (0) | (1) | (2) | (3) | (4) | (5) | (6) | (7) | (8) | (9) | (10) |  |
| --- | --- | --- | --- | --- | --- | --- | --- | --- | --- | --- | --- | --- | --- |
| 1. My religious beliefs guide my health decisions | Strongly disagree |  |  |  |  |  |  |  |  |  |  |  | Strongly agree |
| 1. I live my life according to my religious beliefs | Strongly disagree |  |  |  |  |  |  |  |  |  |  |  | Strongly agree |
| 1. My religion prohibits me from getting vaccinated | Strongly disagree |  |  |  |  |  |  |  |  |  |  |  | Strongly agree |

**Q30. Do you agree with the following statements relating to your community/ government leader(s) (e.g. governors, mayors, councilors etc.)? Please rate each statement on a scale from 0 to 10, with 0 being 'strongly disagree' and 10 being 'strongly agree'.** *Community/ government leader(s) can include village chiefs, community health leaders, residential community leaders, etc.*

|  |  | (0) | (1) | (2) | (3) | (4) | (5) | (6) | (7) | (8) | (9) | (10) |  |
| --- | --- | --- | --- | --- | --- | --- | --- | --- | --- | --- | --- | --- | --- |
| 1. The opinion of my community/ government leader(s) is important to me | Strongly disagree |  |  |  |  |  |  |  |  |  |  |  | Strongly agree |
| 1. My community organizes events promoting health | Strongly disagree |  |  |  |  |  |  |  |  |  |  |  | Strongly agree |
| 1. I actively participate in community events, specific to promoting good/improved health and well-being | Strongly disagree |  |  |  |  |  |  |  |  |  |  |  | Strongly agree |

**Q31. Do you agree with the following statements relating to your influencer(s)?**

**Please rate each statement on a scale from 0 to 10, with 0 being 'strongly disagree' and 10 being 'strongly agree'.**

*Influences can include social media influencer(s), local and international celebrities etc.*

|  |  | (0) | (1) | (2) | (3) | (4) | (5) | (6) | (7) | (8) | (9) | (10) |  |
| --- | --- | --- | --- | --- | --- | --- | --- | --- | --- | --- | --- | --- | --- |
| 1. The opinions of my influencer(s) is important to me | Strongly disagree |  |  |  |  |  |  |  |  |  |  |  | Strongly agree |
| 1. My influencer(s) organizes events promoting good/ improved health and well-being | Strongly disagree |  |  |  |  |  |  |  |  |  |  |  | Strongly agree |
| 1. I actively participate in health-related events hosted by my influencer(s) | Strongly disagree |  |  |  |  |  |  |  |  |  |  |  | Strongly agree |

**Q32. Which of the following channels do you most commonly use when actively looking for health-related information including information about vaccines? Please select all that apply.**

- Search engines (e.g., Google, Yahoo!)
- Social Media (e.g., Facebook, Twitter, TikTok, Instagram, YouTube)
- Messaging platforms (e.g. WhatsApp, Telegram, Facebook Messenger)
- Consumer reviews
- Product and brand websites
- Product and brand blogs/video blogs
- Websites specializing in health-related information (e.g., WebMD, MayoClinic, Hello Health)
- Government or health agency websites or portals (e.g. Ministry of Health, Ministerio de Salud, Ministério da Saúde, Kementerian Kesihatan)
- Patient advocacy websites
- Scientific journals (e.g. PubMed, New England Journal of Medicine)
- Online forums, 'Question & Answer' services (e.g., Quora, Reddit)
- Newspapers, magazines
- Television
- Radio
- Others: ___

**Q33. Out of the following channels that you use, please select the top 3 channels that you most commonly use when looking for health-related information.**

|  |
| --- |

**Q34. Which of the following types of people/organizations do you trust to receive health-related information from?**

- Doctors
- Nurses/ Paramedics
- Pharmacists
- Government
- Religious leaders
- Community leaders (e.g. village chiefs, community health leaders, residential community leaders, etc.)
- Pharmaceutical companies
- Non-profit organizations (e.g. World Health Organization)
- Scientific organizations
- Family, friends and/ or colleagues
- Influencers (e.g. local and international celebrities, etc.)
- Others: ___

**Q35.** **Out of the types of people/organizations that you trust, please select the top 3 sources that you trust the most when looking for health-related information.**

|  |
| --- |
